# Innovative mouse model mimicking human-like features of spinal cord injury: efficacy of Docosahexaenoic acid on acute and chronic phases

**DOI:** 10.1038/s41598-019-45037-x

**Published:** 2019-06-20

**Authors:** Sara Marinelli, Valentina Vacca, Federica De Angelis, Luisa Pieroni, Tiziana Orsini, Chiara Parisi, Marzia Soligo, Virginia Protto, Luigi Manni, Roberto Guerrieri, Flaminia Pavone

**Affiliations:** 1 CNR - National Research Council, Institute of Cell Biology and Neurobiology, 00015 Monterotondo Scalo, Italy; 2 IRCCS - Santa Lucia Foundation, 00143 Roma, Italy; 3 CNR - National Research Council, Institute of Translational Pharmacology, 00133 Roma, Italy; 4 Department of Electrical, Electronic, and Information Engineering «Guglielmo Marconi», University of Bologna, 40136 Bologna, Italy

**Keywords:** Neurodegeneration, Spinal cord injury

## Abstract

Traumatic spinal cord injury has dramatic consequences and a huge social impact. We propose a new mouse model of spinal trauma that induces a complete paralysis of hindlimbs, still observable 30 days after injury. The contusion, performed without laminectomy and deriving from the pressure exerted directly on the bone, mimics more closely many features of spinal injury in humans. Spinal cord was injured at thoracic level 10 (T10) in adult anesthetized female CD1 mice, mounted on stereotaxic apparatus and connected to a precision impactor device. Following severe injury, we evaluated motor and sensory functions, and histological/morphological features of spinal tissue at different time points. Moreover, we studied the effects of early and subchronic administration of Docosahexaenoic acid, investigating functional responses, structural changes proximal and distal to the lesion in primary and secondary injury phases, proteome modulation in injured spinal cord. Docosahexaenoic acid was able i) to restore behavioural responses and ii) to induce pro-regenerative effects and neuroprotective action against demyelination, apoptosis and neuroinflammation. Considering the urgent health challenge represented by spinal injury, this new and reliable mouse model together with the positive effects of docosahexaenoic acid provide important translational implications for promising therapeutic approaches for spinal cord injuries.

## Introduction

According to the World Health Organization (2013) between 250 000 and 500 000 people suffer a spinal cord injury (SCI) every year. People with SCI live with devastating disabilities, experience chronic pain and a significant percentage show signs of depression, causing detrimental effects on quality of life and a considerable financial burden on society. Tissue damage to the spinal cord results in loss of motor and sensory functions and is produced by three main causes: i) destruction from direct trauma ii) compression due to spinal discs or bone material pressing against the cord, and iii) reduction in blood flow from the initial damage i.e. ischemia.

Considering the heterogeneity and the complexity of the phenomenon it is critically important to consider how much the most utilized experimental models mimic processes and consequences occurring in humans. There are a number of models utilized, whose differences are based on the mechanism of injury; the most common surgical models include transection, compression and contusion among others. Everyone shows advantages and disadvantages mainly related to difficulties to truly express human condition^1–6^. In general, total spinal transection that is performed also in large animals and is widely used to assess regeneration, has little clinical relevance, whereas partial transection, even if permits a contralateral side comparison, is limited by an inconsistent reproducibility. On the other hand, the contusion model, considered closer to pathophysiology that occurs commonly in humans, implies laminectomy before the spinal cord contusion that reduces the damage and completely eliminates the compression of bone. In the present paper we present, for the first time, a variation of the spinal cord contusion murine model, in which no laminectomy is performed. This model mimics accurately the mechanical trauma found in a variety of human SCI pathologies, reproducing the variety of pathological features encountered in clinic and inducing a complete absence of motor recovery. We consider our preclinical model a realistic tool for investigating both anti-inflammatory and neuroprotective strategies as a proof of concept for novel treatments.

Although there are differences in spinal cord neuroanatomy, physiology and reaction to injury between animal species and human, most of knowledge derives from animal models. Experimental studies have demonstrated that SCI is a two-step process marked by distinct changes during the primary and secondary injury phases^7,8^. Primary mechanical injury refers to damage sustained at the time of traumatic event through shear forces to axons and/or blood vessels. The secondary phase refers to the body’s response to the primary injury and involves a host of cellular and molecular events that occur immediately after injury and persist for months to years’, with a temporal trend that starts 2 hours after injury (acute), continues with a subacute step (7 to 30 days from injury) and becomes chronic 30 days after injury^8,9^.

Inflammation, which occurs immediately and lasts for several weeks after SCI, plays a fundamental role in the secondary injury phase. The inflammatory response is crucial for the clearance of tissue debris, but at the same time negatively interferes with healthy tissue and exacerbates the damage, being the extent of inflammatory response worsened by proinflammatory cytokines, secreted by microglia and recruited to the injury site, and by immune reactive cells^10,11^.

Wallerian degeneration occurring after SCI is characterized by axonal damage, myelin sheath degeneration and demyelination. Different events are involved including the uncontrolled oxidative stress, ischemia and glutamate-mediated excitotoxicity. A main role is played by oligodendrocytes, the only myelin-forming cells within the central nervous system, highly vulnerable to damage leading to necrosis and apoptosis^12^. In subacute and chronic phases, the formation of glial scar in the surrounding of cystic cavity at the necrotic part of injury epicenter represents a physical barrier to neural function improvement.

Recent papers show that Docosahexaenoic acid (DHA) is able to positively influence neuroinflammation, promotes tissue protection and neuroplasticity, and increases functional recovery^13–20^, even if also modest effects were reported in humans^21^ and it is not clear by which mechanism DHA exerts its action^22^. We though it was important to investigate also in our model the effects of DHA administration and the mechanisms by which this molecule exerts its effects *in vivo*. In the present study we confirmed and extended the beneficial effects of repeated systemic administration of DHA on the primary and secondary phases of SCI, induced in mice by a new experimental procedure that causes human-like functional deficits and tissue/cell response. We demonstrated that DHA treatment fully reinstates motor and sensory function, exerts neuroprotective action, modulates cellular response to injury (apoptosis and survival) and inflammation in astrocytes and microglia, finally resulting in promotion of spinal regeneration.

## Results

### A new mouse model of SCI

**Figure 1:**
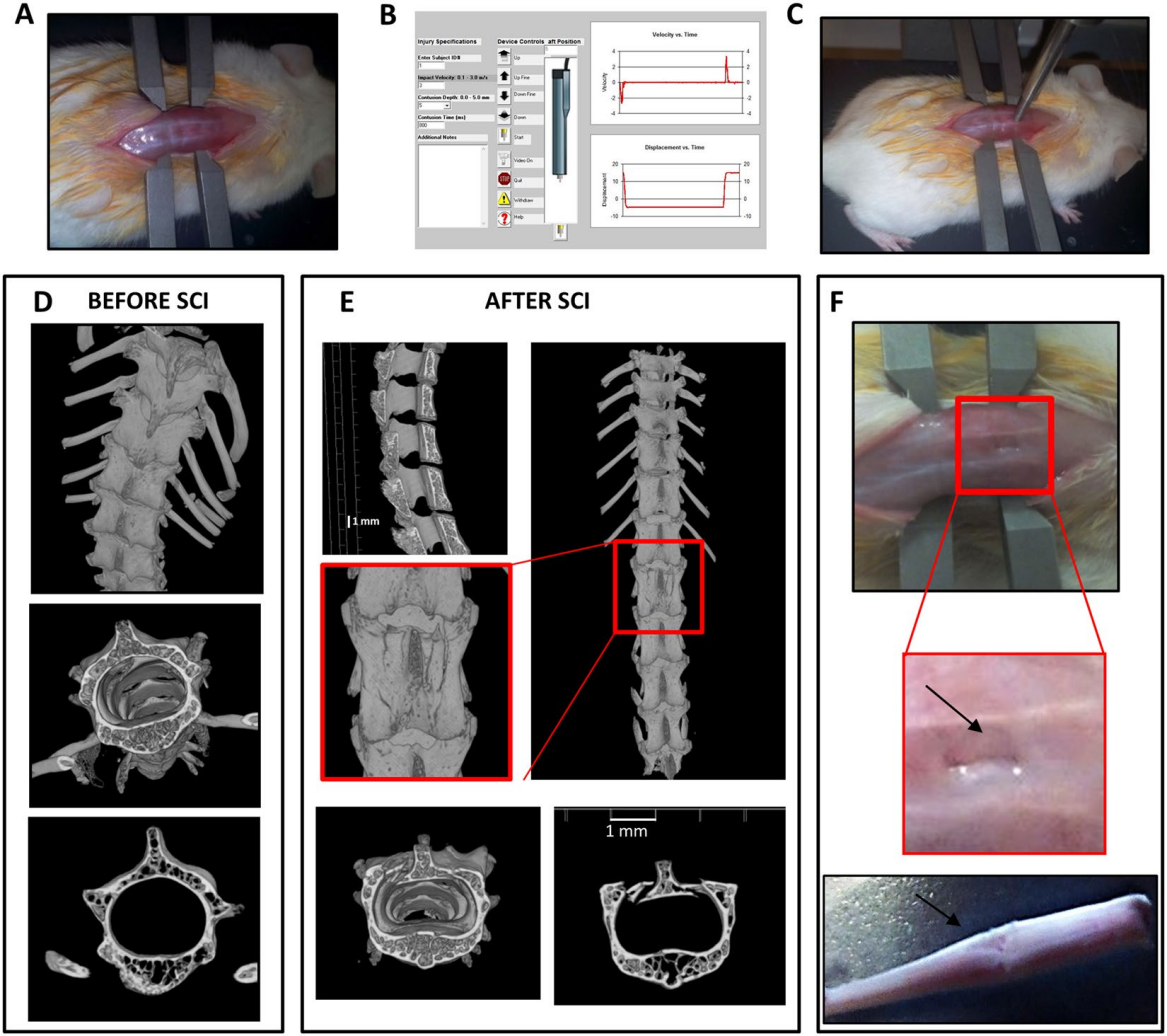
Generation of severe spinal cord injury. (**A**) Representative example of the exposition of the spinal cord in anesthetized animal mounted on a stereotaxic apparatus. (**B**) Spinal adaptors are connected to a cortical PinPoint precision impactor device (Stoelting), being the impactor positioned on T9-T11 vertebrae. The graphical impact parameters are used to identify potential outliers. To obtain a severe spinal trauma the following parameters are set: - middle, round and flat tip (#4); - velocity 3 m/sec; - depth 5 mm; - dwell time 800 ms. (**C**) Representative example of the impactor on the spinal cord of an anesthetized animal. (**D**) Representative longitudinal and cross-sectional μCT images of thoracic spinal cord in intact and (**E**) in SCI mice, magnification of the impact zone at the thoracic level after SCI is shown in the square. (**F**) Impact generates a perforation of vertebrae bones and some fragments are visible (zoom, arrow). After impact spinal cord appears compressed (bottom).

A severe SCI contusion model should mimic the pathological human condition and have clinical translational potential for novel treatments. We present a new methodology for a consistent model of severe SCI, in which no laminectomy is performed. The use of a stereotactic frame and computer-controlled impactor allows for creation of reproducible injury (Fig. 1A–C). The contusion, performed at T9-T11 spinal level (Fig. 1D–F), generated an impairment of the hindlimbs (paraplegia) without involving the forelimbs’ function (Supplementary Video 1, taken at D30 post-injury). As in human spinal trauma, vertebrae of animals subjected to SCI presented several fractures, evidenced by microCT imaging (Fig. 1E). The functional results of this contusion model, examined by means of Basso Mouse Scale (BMS)^23^, demonstrated that different degrees of impact produce significantly different behavioural responses, which may be classified as mild, moderate and severe. We used the following parameters to induce a severe impact: dwell time of 800 ms, velocity of 3 m/sec and depth of 5 mm (for details see Supplementary Table S1).

**Figure 2:**
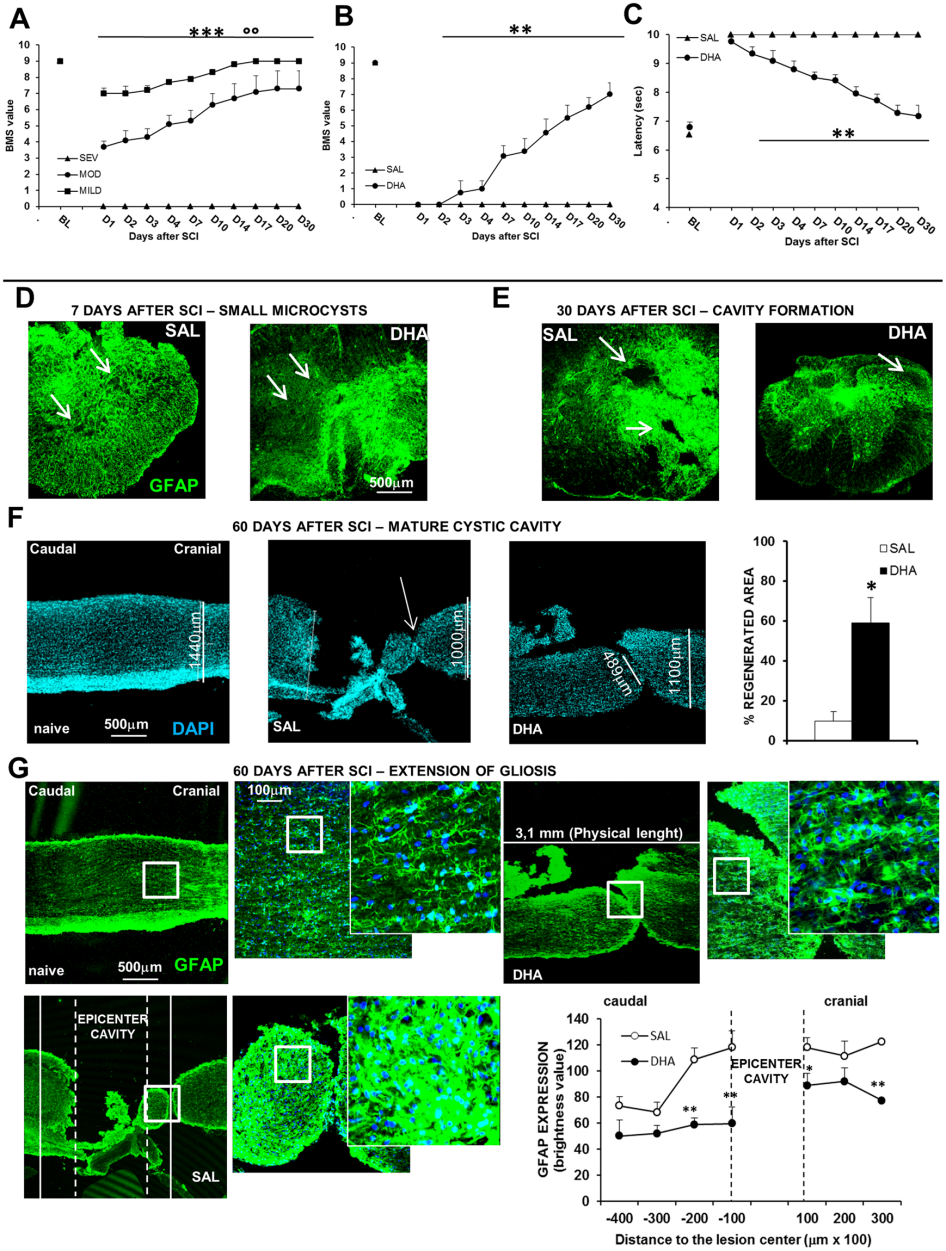
Behavioural responses, microcystic degeneration and cystic cavitation in the new mouse model of SCI. (**A**) BMS scores in female mice subjected to different degrees of impact. BMS defines the locomotion in recovery after SCI. The BMS score ranges from 0 to 9, where 0 indicates complete paralysis and 9 normal movement of the hindlimbs. Motor function recovery is significantly different among the three degree of impact (°°p < 0.01 mild vs moderate; ***p < 0.001 mild and moderate vs severe SCI, Tukey/Kramer; n = 5 mice/group). (**B**) BMS scores and (**C**) thermal threshold in daily ip saline- (SAL) or DHA-injected (from D1 to D5 after SCI) females. DHA-treated animals (n = 8) show a significant improvement in motor function, as revealed by higher BMS values in comparison with SAL-treated animals (n = 11) (***p < 0.001), and a significant recovery of tail-flick reflex (***p < 0.001) (Tukey/Kramer). (**D**) Representative images (5X) of small microcysts, as revealed by GFAP (green) immunostaining, 7 days after SCI in SAL- and DHA-treated mice. (**E**) Representative image (5X) of cavity formation 30 days after SCI in SAL- and DHA-treated mice. (**F**) Nuclei stained with DAPI in longitudinal representative images (5X) of spinal cord in naïve, SAL- and DHA-treated mice, 60 days after SCI; DHA induced significant (**p < 0.01) beneficial effects, as shown by the percentage increase of regenerated area in the graph (n = 3 mice/group). (**G**) GFAP (green) staining in longitudinal representative images (5X) of spinal cord in naïve, SAL- and DHA-treated mice 60 days after SCI. The squares represented high magnification images (20x and zoom 2 respectively). The areas 200 μm caudal and cranial in respect to the epicenter cavity are quantified in the graph. Note, in the graph, that DHA significantly reduced GFAP expression at caudal and cranial level (n = 4 sample/group) (*p < 0.05, **p < 0.01 vs SAL Student’s t test).

Only the severe trauma was able to induce a permanent paraplegia in female mice, which, when subjected to mild or moderate impact, spontaneously recovered (Fig. 2A). ANOVA two-way for repeated measures showed significant main effect for trauma intensity (F_2,12_ = 106,2; p < 0.001), time (F_9,108_ = 25,713; p < 0.001) and a significant intensity × time interaction (F_18,108_ = 7,658; p < 0.001). Male mice were not considered because preliminary experiments have shown that they gradually recovered after SCI (Supplementary Fig. S1). Thus the subsequent experiments were carried out in female mice subjected to severe trauma.

Behavioural, histological/morphological and molecular changes induced by severe contusion were investigated 7, 30 or 60 days after trauma in saline-injected mice, which represented our control group for SCI. The extent and type of physical damage to the spinal cord, which reflect the ensuing level of functional disruption, are impressive in this contusion model. Most of features considered hallmarks in human SCI were analysed. As detected by GFAP staining, small microcysts were present 7 days after SCI (Fig. 2D), followed by cavity formation 30 days after surgery (Fig. 2E) and by extension of cystic cavity (Fig. 2F) and gliosis (Fig. 2G), after 60 days.

The results obtained legitimize the use of CD1 female mice subjected to a severe impact without laminectomy as a clinically relevant mouse model. In the following results section a comparison with DHA-treated animals was performed to evaluate also in our murine model the already reported neuroprotective efficacy of DHA^19^ and to better understand its mechanisms of action.

### SCI-induced paraplegia and absence of tail-flick reflex: reversion by DHA

The first day after surgery DHA- and saline-treated animals did not show significant differences in Basso Mouse Scale (BMS)^23^ scores (Fig. 2B), showing both groups no movement of hindlimbs. However, after a week following surgery, only DHA-treated animals improved hindlimb motor function, with significantly higher open field motor ratings (>3 points on the BMS scale) compared to saline-treated animals. After 4 weeks following surgery, DHA-treated mice completely recovered motor performance while saline-treated mice were still totally paralyzed. ANOVA two-way for repeated measures showed significant main effect for treatment (F_1,18_ = 36,06; p < 0.001), time (F_9,162_ = 24,32; p < 0.001) and a significant severity × time interaction (F_9,162_ = 29,73; p < 0.001). Moreover, after SCI, saline-treated mice did not show tail withdrawal and always reached cut-off latency (10 sec) during TF measurement (Fig. 2C); on the other hand, DHA-treated mice started to react to the thermal stimulus two days after lesion and fully restored the thermal threshold at day 20, as observed in uninjured animals. ANOVA two-way for repeated measures showed significant main effect for treatment (F_1,16_ = 86,07; p < 0.001), time (F_9,144_ = 15,34; p < 0.001) and a significant treatment × time interaction (F_9,144_ = 24,11; p < 0.001).

Sham-operated animals following saline or DHA treatment, tested for BMS and TF reflex, did not show any difference in comparison with naïve mice (Supplementary Fig. S2).

### SCI-induced macroscopic tissue damage: amelioration by DHA

We examined whether the improvement in motor function mediated by DHA treatment was associated with a reduction in primary and secondary spinal injury in comparison with control animals (saline-treated mice) (Fig. 2D–G).

Histological sections of spinal cord stained for GFAP, 7 days after impact, showed a reactive gliosis and the presence of small cavities in saline-injected mice; DHA reduced the mycrocysts (Fig. 2D) as well as cavity formation 30 days after SCI (Fig. 2E). Moreover, 60 days after surgery, DHA-treated mice reduced the cystic cavity, as demonstrated by the significant increase of regenerated area (Fig. 2F; t_10_ = 3,699; p < 0.01).

It is noteworthy to observe the different GFAP expression, which reveals the extension of gliosis, in saline compared to DHA tissue: GFAP staining is highly localized at the epicenter but it is still present along the spinal cord after SCI, while it significantly decreased after DHA treatment not only in proximity of cavity but also at caudal and cranial levels (Fig. 2G). Moreover, as revealed by high magnification images, different degrees of atrogliosis depending on the distance to the epicenter were appreciable. ANOVA two-way for repeated measures showed significant main effect for treatment (F_1,6_ = 12,098; p < 0.05), distance to the epicenter (F_6,36_ = 12,268; p < 0.0001) and a significant treatment x distance interaction (F_6,36_ = 2,492; p < 0.05) (see the graph).

### SCI-induced glial scar formation and microglia activation: reduction by DHA

**Figure 3:**
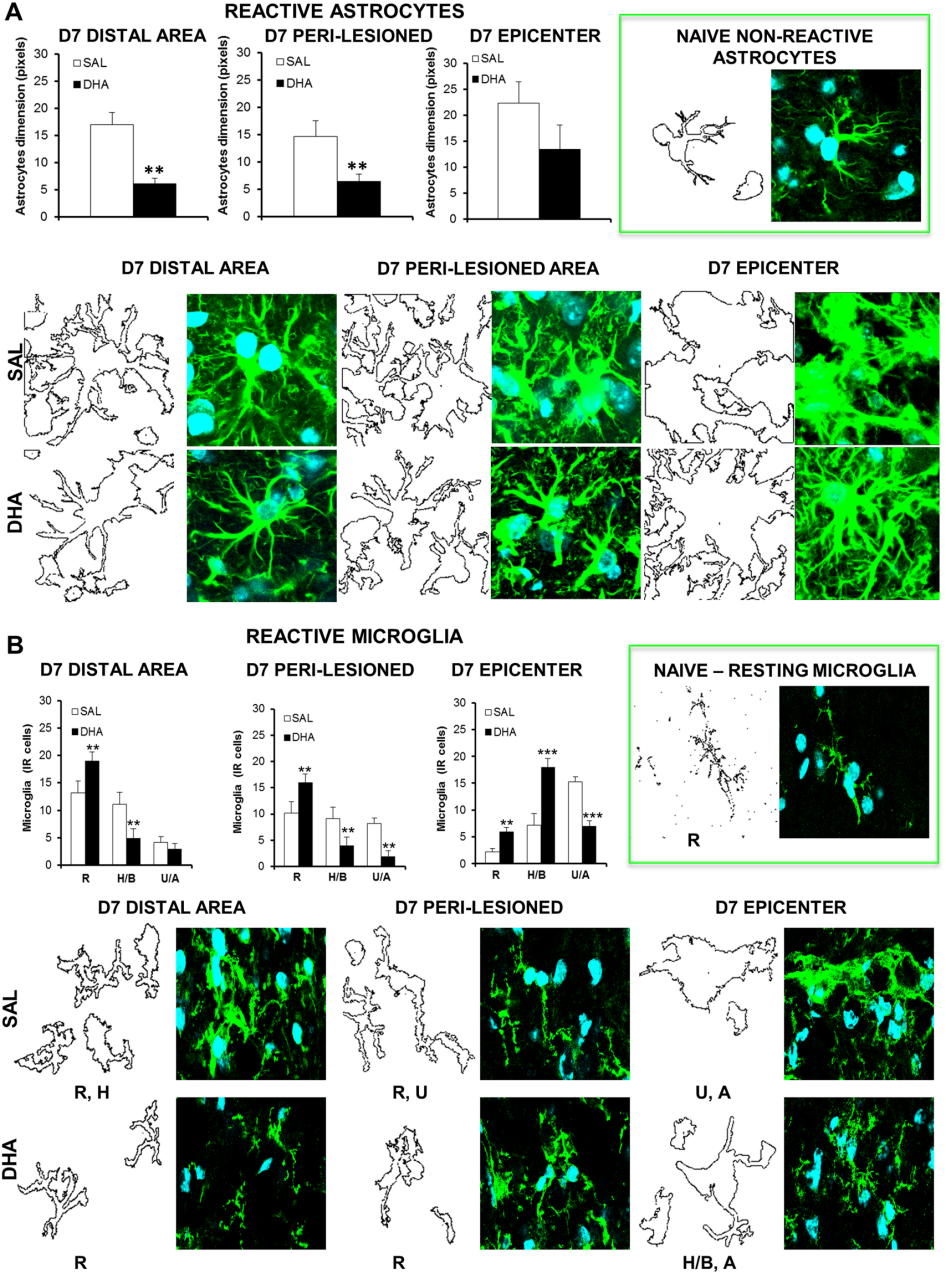
Morphometric analysis of astrocytes and microglia after SCI. Representative high magnification (63X, zoom 3X) images of reactive astrocytes (**A**) and microglia (**B**) at distal, peri-lesioned and epicenter areas of spinal injured tissues, in saline (SAL) and DHA-treated mice, 7 days (D7) after SCI (n = 3 mice/group). Each image was transformed in digital image, where outline of cell silhouettes were identified and automatically measured for astrocytes, while microglia were singularly counted and divided about the different morphology. (**A**) Note the increase of GFAP expression and morphological changes showing hypertrophic status of astrocytes, with glial scar formation in the epicenter area, in comparison with naïve non-reactive astrocytes (in the green frame). As shown in graphs, DHA significantly reduced the dimension of astrocytes (quantified by using RGB method that converted pixel in brightness values) in distal and peri-lesioned areas and, even if not statistically significant, in the epicenter, where also phenotypic changes occurred in comparison with SAL-injected mice. (**B**) After SCI microglia show a hyperactive condition characterized by several phenotypes, differently distributed in the analysed areas: from resting (R) to hypertrophic/bushy (H/B) and un-ramified/amoeboid (U/A) states. As evidenced in graphs, DHA significantly increased the R state in all areas investigated. The increase of the H/B phenotype observed at the epicenter supports phagocytic activity, which may contribute to homeostasis reinstatement. **p < 0.01, ***p < 0.001 vs SAL Student’s t test.

Morphometric analysis revealed that the traumatic impact was able to generate a strong astrocytes and microglia response (Fig. 3A,B), highlighting significant differences between saline and DHA in astrocyte and microglia dimension and morphology. Figure 3A shows that 7 days after injury astrocytes are particularly reactive and hypertrophic in saline mice in both distal and peri-lesioned areas, while the glial scar formation is evident at the epicenter. DHA reduced astrocytes’ activation, as revealed by the significant decrease in distal (t_10_ = −4,428; p < 0.01) and peri-lesioned areas (t_12_ = −2,977; p < 0.05). DHA did not induce significant differences at the epicenter but led to strong morphological changes: astrocytes are highly ramified but do not show the scarring process as observed in saline group.

Usually microglia have a ramified shape in the resting state and react to the spinal insult changing their phenotypic profile. In the distal area 7 days after SCI Resting (R) and Hyperactive/Bushy (H/B) microglia were most abundant; on the other hand, in the peri-lesioned area all the morphological features of microglia coexist: R, H/B and Un-ramified/Ameboid (U/A) (Fig. 3B). The U/A microglia, which is characterized by a phagocytic activity, was mainly present in the epicenter. DHA significantly increased the R microglia in distal (t_12_ = 2,623; p < 0.05) and peri-lesioned (t_12_ = 3,368; p < 0.01) areas and reduced the number of activated microglia (distal: H/B t_12_ = -3,410; p < 0.01; peri-lesioned areas: H/B t_12_ = −3,337; p < 0.01; U/A: t_12_ = −3,129; p < 0.01), while increased R (t_12_ = 5,423; p < 0.001) and H/B (t_12_ = 6,273; p < 0.0001) and decreased U/A (t_12_ = −7,664; p < 0.0001) microglia in the epicenter.

### SCI-induced proteome alterations: modulation by DHA

A comparative proteomic study of the injured spinal cord from SAL- or DHA-treated animals, 7 days after injury, highlighted a panel of proteins differentially modulated in the two groups, including some proteins that characterize human SCI neurodegeneration. In our experiment we could identify 109 proteins, 56 of which were up-regulated and 53 down-regulated in DHA compared to saline group (Supplementary Table S2).

To interpret and understand the results of proteomics data within the contest of this SCI model, we interrogate the search tool Ingenuity® Pathway Analysis (IPA®, QIAGEN Inc., https://www.qiagenbioinformatics.com/products/ingenuitypathway-analysis)^24^ performing a Core Analysis to know which disease and function were mostly associated with our dataset.

Among all Diseases and Biofunction predicted, we focused our attention on Neurological Diseases, where we found a significant association among several proteins up-regulated in SAL, such as Myelin oligodendrocyte glycoprotein (MOG), Myelin proteolipid protein (PLP1), Sodium/potassium-transporting ATPase subunit beta-2 (ATP1B2), Neurofilament medium polypeptide (NEFM) and movement disorders, neurodegeneration and paralysis (Table 1).

**Table 1:** IPA Disease and Biofunction downstream effect analysis.

Diseases and Biofunction	# molecules
**Movement Disorders** **ACAT1**, AHCYL1, **AK1**, ALB, **ALDH5A1**, ATP1A2, ATP1A3, ATP1B1, ATP1B2, **ATP50**, **ATP6V1E1**, Cdc42, **CKB**, CNP, **DPYSL3**, **ENO2**, **ENO3**, **EPB41L3**, **GAPDH**, GNAO1, GPD1, HSP90AA1, **MAPT**, **MBP**, MOG, NDRG2, NEFL, **PEBP1**, **PGK1**, PLP1, **PPIA**, **PRDX2**, RHOG, SLC1A2, **SNCA**, **SNCB**, **SNCG**, **SOD2**, **SYN1**, **TPI1**, **TUBB1**, **MTUBB3**	42
**Neurodegeneration** ATP1B1, CNP, **DPYSL2**. **DPYSL3**, **GAPDH**, **MAPT**, NEFM, PLP1, RAB1A, SLC1A2, **SNCA**, **SNCB**, **SOD2**	13
**Paralysis** ATP1A2, ATP1A3, ATP1B2, HSPD1, **MAPT**, **MBP**, MOG, NEFM, PLP1, SLC1A2, SLC25A4, SNCA, TUBB1, TUBB3	14
**Glyosis** **ALDH5A1**, **MAPT**, MOG, PLP1, **S100B**, **SNCA**, **SOD2**	7

The table summarizes the predicted relation between molecules modulated in our datasets and biologica function or disease strictly related to SCI. Proteins more espressed in SAL (underlined) and in DHA (bold) spinal tissues are reported.

Several Canonical Pathways were also interestingly associated with our datasets (Table 2). Glycolysis and gluconeogenesis, metabolic pathways that guarantee production and utilization of glucose, essential under all condition of growth, were up-regulated by DHA administration as well as Semaphorin Signaling in neurons. Semaphorins are a family of growth cone guidance molecules implicated in mediating repulsive guidance during neurodevelopment and neuronal regeneration. Interestingly also the Mitochondrial Dysfunction pathway was affected in this experimental model, underlining the relevant impact of SCI on mitochondrial redox states and energy machinery, closely linked to cell death. Specifically, the up-regulation of some peroxiredoxins, of Voltage-dependent anion-selective channel 1 (VDAC1) and several Na^+^/K^+^-transporting ATPases observed after DHA revealed the potential antioxidant properties of this compound, which can exert a neuroprotective action. Moreover, according to the modulation of the protein expression measured by the proteomic analysis, IPA Upstream Regulator Analysis identified upstream regulators that may be responsible for ongoing gene expression changes observed in experimental datasets, helping the comprehension of biological processes occurring in this model. IPA prediction showed that up-regulation of several proteins in DHA treated animals, e.g, superoxide dismutase 2 (SOD2), ATP synthase subunit alpha and beta (ATP5A, ATP5B), can lead to the activation of Insulin-like growth factor 1 receptor (IGFR), Peroxisome proliferator-activated receptor gamma (PPARG1A), Insulin receptor (INSR) and transcriptional regulators involved in the regulation of energy metabolism pathways (Supplementary Fig. S3). These observations support the hypothesis that DHA treatment can favour the injured tissue regeneration.

**Table 2:** Top Canonical Pathways.

Name	p-value	Overlap
Glycolysis I **ALDOA**, **ALDOC**, **ENO2**, **ENO3**, **GAPDH**, **PGAM1**, **PGK1**, **PGK2**, **TPI1**	4.21E-15	39.1% (9/23)
Gluconeogenesis I **ALDOA**, **ALDOC**, **ENO2**, **ENO3**, **GAPDH**, **MDH2**, **PGAM1**, **PGK1**, **PGK2**	6.71E-15	37.5% (9/24)
Semaphorin Signaling in Neurons **CRMP1**, **DPYSL2**, **DPYSL3**, RAC1, RHOG, RHOJ, RHOQ	1.76E-08	13.7% (7/51)
Mitochondrial Dysfunction **ATP5B**, **ATP5D**, **ATP5H**, **ATP5O**, **PRDX3**, **PRDX5**, **SNCA**, **SOD2**, **VDAC1**, VDAC2	3.06E-08	6.3% (10/159)

Proteins more espressed in DHA (bold) and in saline (underlined) spinal tissues are reported.

### SCI-produced myelin alterations: counteraction by DHA

**Figure 4:**
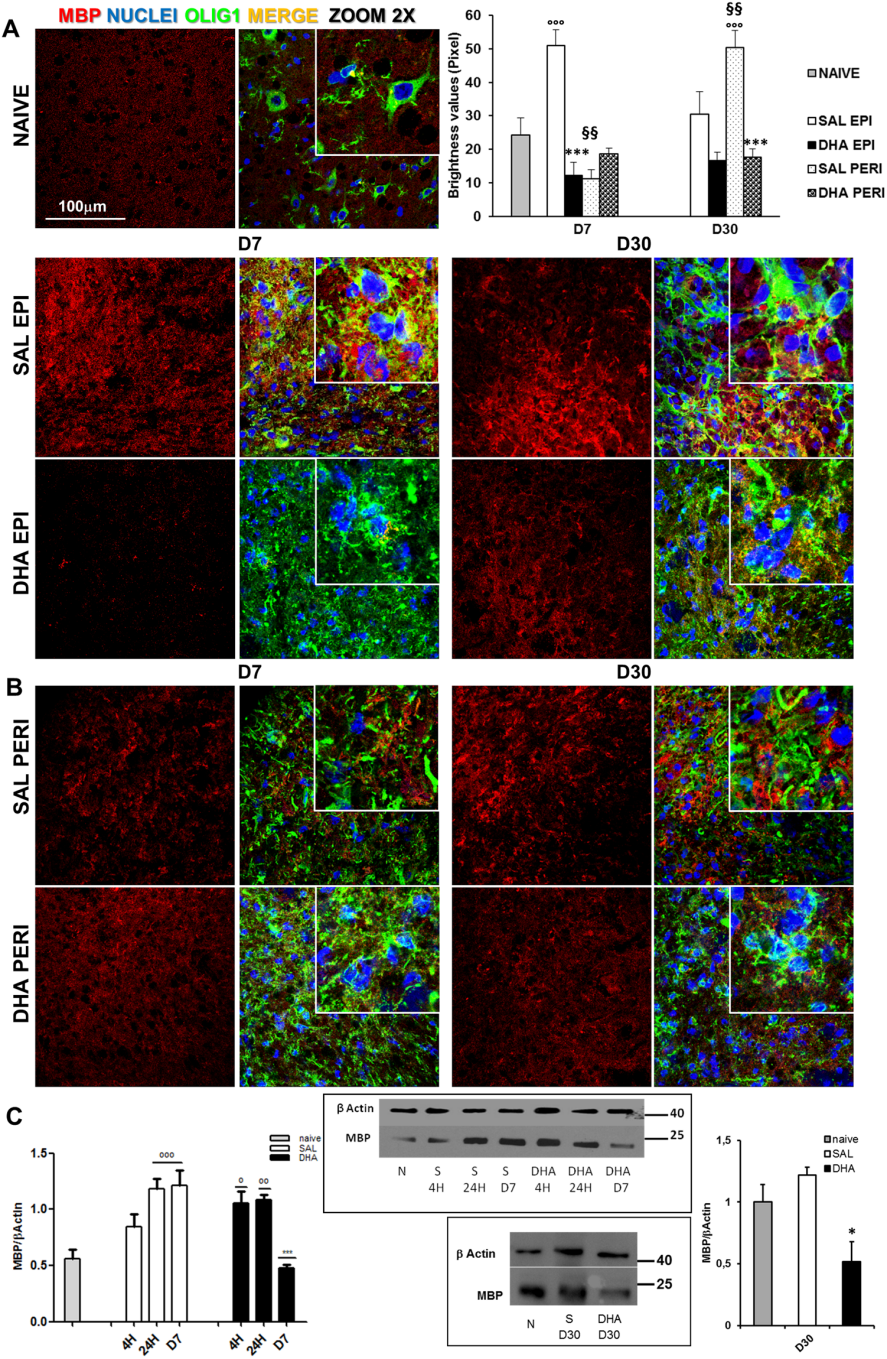
Myelination after SCI: Expression of Myelin Basic Protein. (**A**) Representative images (63X, zoom 2) of MBP expression in naïve and saline (SAL)- and DHA-treated mice at the epicenter (EPI) and (**B**) peri-lesioned (PERI) areas, 7 (D7) and 30 days (D30) after SCI. The quantification of MBP, in graph, showed significant increase of MBP expression in SAL EPI at D7 and SAL PERI at D30 in comparison with naïve, while DHA significantly differed from SAL (n = 3 mice/group) (°°°p < 0.001 vs naïve; ***p < 0.001 vs SAL; ^§§^p < 0.01 SAL PERI vs SAL EPI. Tukey/Kramer). (**C**) Western blot analysis and quantification of MBP in naïve/N, saline (S)(SAL) and DHA-treated mice 4 hours (4 H), 24 hours (24 H) and 7 days (D7) after SCI. The time dependent increase of MBP in injured tissues of SAL mice was gradually reduced in DHA-treated animals, where the expression of MBP reached the naïve values at D7. At D30 (graph on the right) MBP expression in DHA is significantly reduced compared to SAL-treated samples (n = 3 mice/group/time point). (°p < 0.05, °°p < 0.01, °°°p < 0.001 vs naïve; *p < 0.05, ***p < 0.001 vs SAL; Dunn’s Test).

An increase of MBP expression, analysed by immunofluorescence analysis, was observed 7 days after SCI at the epicenter of saline- but not DHA-treated spinal tissues in comparison with those from naive, while in the peri-lesioned areas no differences were evident among groups (Fig. 4A,B). When the myelin protein was analysed at the epicenter 30 days after injury, the immunostaining of saline group appeared enhanced even if not quantitatively significant; a significant increase was observed only in peri-lesioned area of saline group. The samples from DHA-treated group appeared similar to those of naïve group. The analysis of variance (ANOVA one-way) demonstrated significant differences among groups for D7 and for D30 (F_4,24_ = 17,744; p < 0.0001 and F_4,25_ = 8.74; p = 0.0001, respectively).

Investigating by means of Western Blot the time course of MBP expression from 4 h to D7 after lesion in saline and DHA samples in comparison with naïve (Fig. 4C) we confirmed a significant difference among groups (H_6_ = 14,771; p < 0.05, Kruskal-Wallis). We noticed a gradual enhancement of the protein starting 4 h after injury in saline in comparison with naïve samples, increase that reaches a significant effect 24 h and 7 days after injury. Differing from what observed in saline group, the significant increase (p < 0.05), in comparison with the naïve level, observed 24 h after trauma in spinal cord of DHA-treated mice disappeared after 7 days, resulting significantly different (p < 0.01) from the corresponding value of saline group. Further WB analysis 30 days after trauma revealed a significant difference among groups (H_2_ = 6,489; p < 0.05, Kruskal-Wallis), with a significant reduction of MBP in DHA- compared to saline-treated samples.

### SCI-induced up-regulation of growth factors: modulation by DHA

Considering the increased vascular permeability induced by spinal injury and the role of growth factors (GFs) in cell proliferation and differentiation, we examined the implication of some GFs in our SCI model and the effects of DHA on their expression. We investigated a mouse panel (10 total mediators in one experiment) of trophic factors in spinal cord lysates 24 h and 7 days (D7) after SCI. Table 3 shows the significant decreased/increased levels of trophic factors after SCI in tissues derived from saline or DHA mice. Data are shown as fold change (SAL24h/Naïve; DHA24h/ Naïve; SALD7/Naïve; DHAD7/ Naïve;). Any ≥1. 5-fold increase or ≤0.65-fold decrease in signal intensity for a single analyte between samples may be considered a measurable and significant difference in expression. All trophic factors analysed were significantly up-regulated after spinal contusion in the saline group compared to the Naïve condition. In particular, there was a great increase of the basic fibroblast growth factor (bFGF), the Insulin-Like Growth Factor I (IGF-I), the epidermal growth factor (EGF) and the vascular endothelial growth factor (VEGF). These GFs are still up-regulated after DHA treatment but definitely to a lesser extent.

**Table 3:** Mouse Antibody Array Glass Chip: Expression of GFs in spinal cord tissue lysates 24 h and seven days (D7) post SAL and DHA treatment.

Growth Factor	Function	SAL 24 h/Naive	DHA 24 h/Naive	SAL D7/Naive	DHA D7/Naïve
bFGF	basic fibroblast growth factor, present in the subendothelial extracellular matrix of blood vessels and involved in angiogenesis process	15,01 ± 1,56 *p* = *0*,*0007*	2,04 ± 0,29 p = 0,0144	11,26 ± 2,84 p < 0.0001	2,89 ± 0,96 *p* = *0*,*0198*
EGF	epidermal growth factor, plays an important role in regulating the growth, proliferation and cellular differentiation by binding to its receptor EGFR	2,10 ± 0,25 *p* = *0*,*0163*	1,8 ± 0,30 p = 0.0269	2,67 ± 0,82 *p* = *0*,*0139*	2,65 ± 0,37 *p* = *0*,*0037*
IGF-I	insulin-like growth factor I, produced primarily by the liver as an endocrine hormone as well as in target tissues in a paracrine/autocrine fashion. Production is stimulated by growth hormone (GH)	6,46 ± 1,33 *p* *<* *0*,*0001*	1,71 ± 0,44 *p* = *0*,*0589*	8,13 ± 1,55 *p* *<* *0*,*0001*	1,97 ± 0,93 *p* = *0*,*0678*
IGF-II	insulin-like growth factor II, believed to be a major fetal growth factor in contrast to Insulin-like growth factor 1, which is a major growth factor in adults	2,25 ± 0,63 *p* = *0*,*0152*	3,79 ± 1,56 *p* = *0*,*0468*	3,93 ± 1,89 *p* = *0*,*1044*	3,73 ± 0,43 *p* = *0*,*021*
VEGF-A	Vascular endothelial growth factor A, dimeric glycoprotein that plays a significant role in neurons and is considered to be the main, dominant inducer to the growth of blood vessels	1,54 ± 0,46 *p* = *0*,*4027*	1,76 ± 0,48 *p* = *0*,*0598*	1,93 ± 0,60 *p* = *0*,*066*	2,09 ± 0.56 *p* = *0*,*0192*
VEGF R1	Vascular endothelial growth factor Receptor 1, thought to modulate VEGFR-2 signaling and to act as a dummy/decoy receptor, sequestering VEGF from VEGFR-2 binding	3,89 ± 0,81 *p* = *0*,*0002*	1,75 ± 0,43 *p* = *0*,*0474*	2,55 ± 0,90 *p* = *0*,*0426*	2,01 ± 0,55 *p* = *0*,*0268*
VEGF R2	Vascular endothelial growth factor Receptor 2, mediates almost all of the known cellular responses to VEGF	2,27 ± 0,36 *p* = *0*,*0061*	1,74 ± 0,36 *p* = *0*,*0347*	2,69 ± 1,16 *p* = *0*,*1113*	2,39 ± 0,57 *p* = *0*,*0048*
VEGF R3	Vascular endothelial growth factor Receptor 3, mediates lymphangiogenesis in response to VEGF-C and VEGF-D.s	1,87 ± 0.15 *p* = *0*,*0484*	1,29 ± 0,15 p = 0,3351	2,58 ± 1,18 *p* = *0*,*1555*	3,21 ± 0,52 *p* = *0*,*0009*
VEGF-D	Vascular endothelial growth factor D, needed for the development of lymphatic vasculature surrounding lung bronchioles.	1,90 ± 0,32 *p* = *0*,*0185*	1,66 ± 0,32 *p* = *0*,*0485*	1,81 ± 0,60 *p* = *0*,*1152*	1,89 ± 0,47 *p* = *0*,*0273*
HGF	Hepatocyte growth factor, is secreted by mesenchymal cells and acts as a multi-functional cytokine on cells of mainly epithelial origin. HGF plays a central role in angiogenesis, tumorogenesis, and tissue regeneration.	2,54 ± 0,52 *p* = *0*,*0022*	2,05 ± 0,51 p = 0,0161	2,16 ± 0,60 *p* = *0*,*019*	2,20 ± 0,46 p = 0,0059

Mean ± SE of the fold change values (n.3 values for each time point). P values were obtained with a Student’s t-test analysis.

### SCI-induced apoptosis in neurons and oligodendrocytes: reduction by DHA

**Figure 5:**
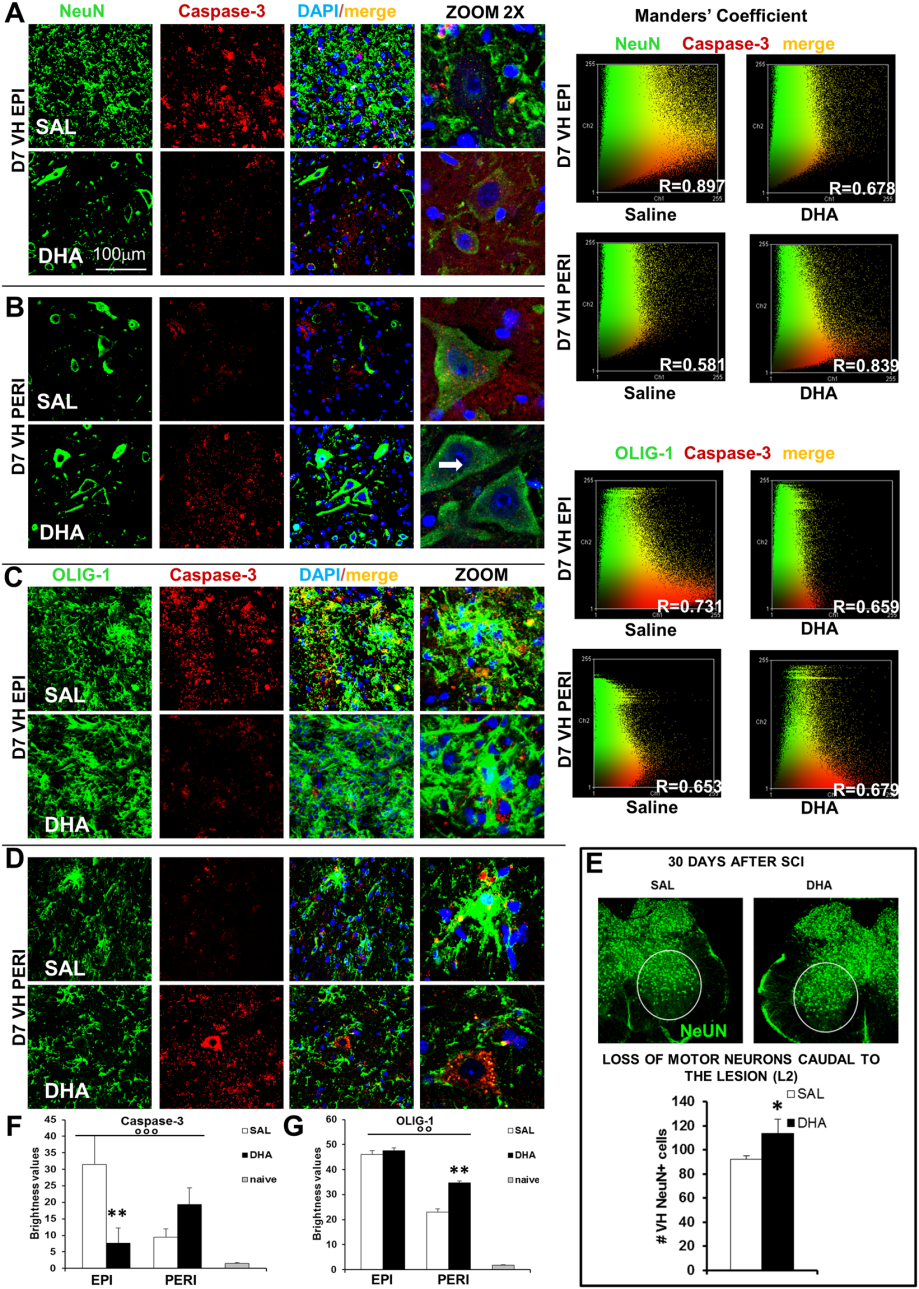
Apoptosis in primary injury phase. Representative examples of high magnification (63X, zoom 2) confocal images of Neurons (NeuN, **A** and **B**) or oligodendrocytes (OLIG1, **C** and **D**) (green), Caspase-3 (red) and their colabeling in ventral horns (VH) of injured spinal cord from saline- (SAL) or DHA-treated animals, 7 days after SCI, at the epicenter (EPI) and peri-lesioned (PERI) areas. DAPI was used to stain nuclei. Manders’ coefficient was utilized to evaluate co-staining between NeuN+ or OLIG-1+ cells and Caspase-3: the R value approximating +1 or −1 indicates linear correlation and 0 indicates absence of correlation. (**E**) Representative images of NeuN positive cells (green) and quantification of neurons (n = 3 mice/group) in ventral horn (VH NeuN+ cells) 30 days after SCI: DHA significantly (p < 0.05; Student’s t-test) counteracted neuronal loss. (**F**) Brightness values of Caspase-3 expression at the epicenter and perilesioned areas in ventral horns of spinal cord from naïve, SAL- and DHA-treated mice (n = 3 mice/group). In comparison with naïve animals, SCI induced a higher expression of Caspase-3 at the epicenter, significantly reduced by DHA treatment. After SCI Caspase-3 expression was less enhanced in the perilesioned area of SAL-treated animals, while a higher expression was present in DHA-treated mice. (**G**) Brightness values of OLIG-1 expression was significantly enhanced after SCI in ventral horns of spinal cord from both saline- and DHA-treated mice, mainly at the epicenter but also in perilesioned area, in comparison with naïve (n = 3 mice/group). °°p < 0.001, °°°p < 0.0001 vs naïve; **p < 0.001 vs SAL. Tukey/Kramer).

To evaluate the level of cell death induced by SCI and to investigate whether DHA treatment interferes with the activation of apoptosis pathways, we detected caspase-3 in grey matter 7 days after SCI, at both epicenter and peri-lesioned zone, by using immunofluorescence analysis. Staining of transverse sections with NeuN (neurons) (Fig. 5A,B,F) or OLIG1 (oligodendrocytes) (Fig. 5C,D,G) and their co-staining (Manders’ coefficient) with caspase-3 revealed a number of pathological changes occurring after contusion. Evident marks of cell death such as destruction and collapse of cell bodies at the epicenter were appreciable in saline group, in both neurons and oligodendrocytes, while DHA treatment preserved some cells. Co-localization analysis revealed a strong correlation (R) between NeuN or OLIG-1 and Caspase-3 expression in the impact zone of SCI saline mice while a modest correlation was present in DHA-treated mice, which instead showed a greater correlation in peri-lesioned area. Neuronal loss in saline-treated mice is still evident in ventral horns at the caudal level (L2) 30 days after surgery (Fig. 5E), time point in which the preventive effect of DHA is also confirmed, as revealed by statistical analysis (t_17_ = 2,192; p < 0.05). ANOVA one-way revealed a significant difference in the expression of caspase-3 after SCI, examined in treated and naïve samples at the epicenter (F_2,24_ = 8,910; p < 0.01) and peri-lesioned area (F_2,24_ = 7,902; p < 0.01). Caspase-3 was significantly higher (p < 0.001) in saline compared to naïve but DHA treatment significantly (p < 0.01) reduced apoptosis at the epicenter in comparison with saline (Fig. 5F). As far as the peri-lesioned area is concerned, treated groups maintained significant differences from naïve but did not significantly differ between them. Figure 5G shows that OLIG-1 was significantly enhanced after SCI both in saline- and DHA-treated mice in comparison with naïve, mainly at the epicenter. ANOVA one-way showed significant difference among groups (F2,24 = 70,657; p < 0.0001 and F2,24 = 57,598; p < 0.0001, for epicenter and peri-lesioned area respectively). In the peri-lesioned area a significant difference (p < 0.001) between saline and DHA groups also emerged. Immunohistochemical analysis did not show any difference in sham-operated animals (saline or DHA) compared to naïve mice (Supplementary Fig. S2).

By means of WB analysis we also investigated p75 neurotrophin receptor (p75NTR), directly involved in apoptosis induced by injury in spinal oligodendrocytes through a JNK-mediated mechanism. We observed that p75NTR and phosphorylated (p)-JNK were increased after SCI in the three spinal levels examined and were down-regulated by DHA, which produced an almost complete disappearance of p75NTR and a parallel great reduction of pJNK (Supplementary Fig. S4).

## Discussion

The results of the present study have important basic and translational potential for the treatment of SCI, whose consequences for patients are terrible, being their quality of life substantially jeopardized. We show that DHA exerts a long-lasting neuroprotective action and a significant improvement of functional recovery in a new contusion animal model, which mimics the dramatic human condition.

Considering the complexity and difficulties involved in translating findings from animal models to clinical trials for this pathological state, it is substantial to choose a model as valid as possible as starting point for preclinical evaluation of pharmacological treatments.

There are several animal models used to induce a lesion; they include contusion, hemisection or complete transection, and compression models^4,6^. Lesions induced by these methods may be reproducible; however, they are unable to reproduce the biomechanical compression and the systemic complications associated to human injuries. There are several aspects on which the choice of animal model may depend. As reported in a survey of physicians and scientists involved in the field of neurotrauma^25^, the contusion model was universally accepted to be clinically relevant, being contusive in nature the majority of SCI in humans. Differing from the transection model that induces only focal tissue pathology limited to the epicenter, the contusion induces a widespread tissue pathology that extends beyond the epicenter of injury^26–29^. The contusion model may present the difficulty to distinguish between spared and regenerated axons, but may represent, besides its clinical relevance, a strong advantage for the study of the pathophysiology of SCI^30^.

The high individual variability, among strains and species, further highlights the difficulties to translate experimental findings and the importance of the appropriate model^23,31–34^. It has been reported that rats but not mice develop cystic cavity^30^; however, this is mainly true for C57BL/6 strain, while CD1 strain, as shown in this study, presents cavitation even if to a lesser extent than rats^35^.

To uniform the force delivery across the spinal cord volume, we utilized the computer-controlled impactor stabilizing the spine before impact^36^. Furthermore, to more accurately reproduce a contusive injury as in humans, we decided to introduce in our animal model a new important feature, namely the absence of laminectomy. This peculiarity is what specifically characterizes this compared to other contusion models and permits us to include and examine also the additional damage derived from bone destruction and its consequences.

The experimental contusion model described here causes in female mice a permanent paralysis with evident hindlimb deformity and loss of tail-flick reflex and presents from histopathological and morphological point of view the devastating characteristics of primary and secondary lesion induced by spinal injury, as previously described in literature^11,37,38^. We observed pathological changes such as cystic cavitation, demyelination, neurons and oligodendrocytes apoptosis, loss of motor neurons, together with proliferation and activation of microglia and astrocytes, accompanied by inflammatory and immune responses. As a matter of fact, intense local inflammation is an immediate consequence of injury, which causes progressive cavitation and exacerbates the extent of the primary lesion; resident astrocytes become reactive, undergo hypertrophy and form a dense glial scar. On the one hand the glial scar facilitates the restoration of the blood-brain barrier integrity, on the other constitutes a barrier that impedes the axon regeneration and the connections necessary for the functional recovery^39^.

We observed that the reactive astrocytes, which migrate toward the lesion site, display hypertrophy and enlarged cell somas not only at the epicenter but also in the peri-lesioned and distal areas. Morphological shifts were evident also for microglia: these cells move from the resting highly branched state to an unramified/amoeboid hypertrophic state, mainly in lesion epicenter, suggesting phagocytic activity and pro and anti-inflammatory cytokines release.

In the last decade there was much debate on the anti-inflammatory, neuroprotective and pro-regenerative action of omega-3 fatty acids, mainly DHA, in different neurodegenerative disorders^15,40^. Experimental evidence suggests its effect on spinal cord lesions in animal models^16,19^, while its potential benefit in regenerative medicine is still uncertain and modest effect is also reported^21^. Some studies reported improved outcome after acute administration of DHA alone^13,17,18^ or in combination with a DHA-enriched diet^14,17,41^ or with rehabilitation^20^ therapy. However, most researches focused on the acute phase of spinal trauma without taking into account the most deleterious second phase. Differing from previous studies, we evaluated the effects of repeated systemic administration of DHA, daily for 5 days, on both acute and intermediate/chronic phases. Moreover, the papers examined, although supporting the use of DHA as possible therapy in spinal trauma, utilized a mouse or rat SCI model characterized by spontaneous restoration of motor function also in vehicle treated-animals. Our model, in which no improvements are observed even 30 days after injury, represents an encouraging challenge to deeply investigate the therapeutic action of this substance in a clinical perspective. We observed that after DHA administration mice had retrieved in few days the ability to walk and the withdrawal reflex to a thermal stimulus. This impressive functional improvement indicates a regenerative potential of DHA, encouraging further investigation on mechanism of action of primary and secondary phases. The investigation of the progression of the pathology from the epicenter and peri-lesioned area to the distal site may provide extensive evidence for long-lasting benefits in contusion-induced SCI.

The dramatic changes elicited by spinal injury to vascular networks and neural architecture, characterized by local inflammation, progressive cavitation and glial scar formation, were significantly affected by DHA. Seven days after impact the treatment reduced SCI-induced up-regulation of GFAP, a hallmark of reactive astrocytes, as well as the presence of small microcysts. Interestingly, when the gliosis was observed 30 and 60 days following the damage, the decrease was evident not only in the proximity of the cavity but also at caudal and cranial levels and is associated to tissue regeneration. DHA reduced glial scar after SCI, suggesting that it creates a permissive environment for axonal growth and myelination and contributes to functional recovery. In addition, it is noteworthy that after DHA some morphological changes occurred, both in astrocytes and microglia. Astrocytes were less reactive and did not show the scarring process. Importantly, also microglia activation was significantly reduced by DHA: morphological analysis showed evident changes in phenotype, with a strong increase of the R shape in all zones examined at D7 and a reduction of U/A at the epicenter in comparison with saline group.

The proteomic analysis further supports our model and the complexity of pathological events that contribute to the lack of functional recovery: SCI significantly modulates the expression of many proteins. It is particularly interesting to note that, among the Disesases and Biofunction strictly related to SCI and predicted by IPA analysis of the proteomics data, most of the proteins associated with paralysis were up-regulated in SAL. Moreover, SCI (saline mice) induced a strong up-regulation of two myelin proteins: Myelin-oligodendrocyte glycoprotein (Mog) and Myelin proteolipid protein (Plp1), which were not induced after DHA administration. It is reported that the overexpression of Mog, a kind of myelin-derived inhibitor, strongly inhibits axonal regeneration^42^, while Plp1 induces demyelination and inflammation throughout microglia activation^43^.

Considering the long-term care of paraplegic patients, great attention should be also paid to metabolic anomalies and mitochondrial dysfunction, typically occurring during the secondary injury phase. These alterations can expose individuals to higher risk of diseases, therefore their control could represent a potentially therapeutic strategy^44,45^. Then, among all pathways modulated, we focused on metabolic pathways, such as glycolysis and gluconeogenesis, and on mitochondrial functionality, whose central role in the regulation of cell life and death is well known^46^. In tissues from DHA-treated mice characteristic proteins of these pathways resulted up-regulated compared to saline, e.g. ATP synthase subunits, superoxide dismutase or VDAC, one of the mitochondrial proteins controlling cross-talk between mitochondria and the rest of the cell^47^.

Reaction to SCI leads to strong up-regulation of different GFs. Specifically, although the most part of GFs analyzed are similarly and significantly expressed both in saline and in DHA, bFGF, IGF-I and VEGF-RI are particularly up-regulated in saline-SCI mice, presumably for the need to face a major loss of neurons. It has been demonstrated that the potent angiogenic and endothelial cell mitogen factor bFGF is strongly up-regulated for all the time of oligodendrocytes precursor cells (OPCs) proliferation and maintains OPCs in a continual mitotic renewal state^48^. OPCs proliferation and oligodendrocytes survival are also promoted by IGF-I. In accordance with the literature, we found a pronounced oligodendrocytes expression after injury in the glial scar region, and, considering the ability of astrocytes to secrete bFGF and IGF, we suggest astrocytes as source of influence for survival and proliferation of OPCs. In addition, although controversial reports exist on its role^45,49^, also VEGF is released by astrocytes and is crucially involved in mediating vascular changes following different types of injury^50^.

Seven days after injury, there is widespread demyelination of spared axons that is associated with additional loss of axons. Since massive oligodendrocyte death attributable to apoptosis occurs acutely after SCI, it is likely that endogenous OPCs are unable to completely restore the loss of myelin in SCI. Necrotic and apoptotic cell death signaling events implicate the activation of caspases in injury-induced neurodegeneration and functional impairment. Caspases are activated early after injury in neurons and oligodendrocytes as well as in some astrocytes; caspase-3 is also activated in gray and white matter, several segments rostral or caudal to the impact^51^. Our data are consistent with previous observations suggesting how Caspase-3 differentially contributes to the expansion of the lesion and to grey matter damage after SCI. Caspase-3 activation could be also related to the increased expression of pJNK and p75NTR proteins, demonstrated in saline-treated mice 7 days after SCI in VH spinal epicenter and distal area. These markers may therefore reflect one step in a general cell death pathway leading either to necrosis or to apoptosis, depending on available energy^52,53^. DHA treatment was able to drastically reduce Caspase-3, pJNK and p75NTR mainly at the epicenter, 7 days after SCI.

These results are corroborated by the observation that in spinal cords of DHA-treated mice, besides oligodendrocytes, a major number of intact neurons cell bodies were present, indicating an elevated neuroprotective action.

## Conclusions

In summary, we developed in mice a new, reliable and sensitive animal model that induces severe spinal injury with long-lasting functional deficits. The model presented in this study can not be representative of all spinal cord injuries since in human population SCI is extremely heterogeneous; nevertheless it is particularly relevant for its capacity to mimic not only paralysis but also several histological features and pathophysiological aspects observed in human pathology, allowing the investigation of translational potential of pharmacological therapies for traumatic SCI. The positive effects of DHA showing capacity of controlling gliosis and apoptosis in paraplegic mice encourage the use of this compound as a therapeutic tool for neuroprotection after SCI.

## Material and Methods

### Animals

CD1 female mice (Charles River Labs, Como, Italy and CNR-EMMA facility, Monterotondo, Italy) weighing about 30–35 g at the beginning of the experiments were used. Male mice were not considered because preliminary experiments have shown that they gradually recovered after SCI (Supplementary Fig. S1). Upon their arrival in the laboratory (at least 2 weeks before the experiments), mice were housed as previously described^54^, in accordance with the guidelines of the Committee for Research and Ethical Issues of IASP (PAIN^®^ 1983, 16, 109–110) and of the European and Italian National law (DLGs n.26 del 04/03/2014, application of the European Communities Council Directive 2010/63/UE) on the use of animal for research. The experimental protocol was approved by the Italian Ministry of Education, Universities and Research (Protocol n. 33/2014).

### Surgery

To perform SCI, animals were deeply anesthetized with a mixture 1:1 of Rompun (Bayer 20 mg/ml; 0,5 ml/kg) and Zoletil (100 mg/ml; 0,5 ml/kg), the back skin was shaved and disinfected with betadine and an incision to expose the vertebral column at the thoracic level was made. Animals mounted on a stereotaxic apparatus connected to a cortical PinPoint Precision Impactor Device (Stoelting) (https://www.stoeltingco.com/cortical-pinpoint-precision-impactor.html) were maintained at 37 °C throughout surgery. The system is designed to provide the user with precision control, power, and flexibility to generate accurate, reliable, and reproducible results. To obtain different degree of trauma severity we set up parameters, as described in Supplementary Table S1. Preliminary experiments were carried out in female mice to establish the right parameters inducing a severe SCI, on the basis of the BMS^23^ and of the nociceptive sensitivity measured by Tail-flick test (TF). It has to be considered that some variability is observed and not all animals subjected to “severe” parameters react with a complete paralysis. Probably a different pressure of the bone on the spinal cord could be in part responsible of this variability, as occurs also in humans. We included only mice that reached 0 value in the BMS.

A severe spinal trauma was induced on the basis of the following parameters: middle, round and flat tip (#4); velocity 3 m/sec; depth 5 mm; dwell time 800 ms. Spinal cord was injured at thoracic level 10 (T10) and no laminectomy was performed, as previously described^55^, thus the contusion deriving from the pressure exerted on the bone resembled more closely to human traumatic injury (Fig. 1).

Analysis of the graphical impact parameters, operated by the PinPoint software, was used to identify potential outliers. Behavioural analyses were used to corroborate differences in injury severity.

Careful monitoring of animals with daily weight recording and bladder expression allowed the early detection of post-operative complications. As postoperative care, the bladder was compressed by manual abdominal pressure twice/daily until bladder function was restored. Animals were maintained at 37° with warmed pad in the first 24 h, rehydrated with 1 ml of ringer lactate and, to support nutrition, humid food was inserted in the cage. Mice used and mortality are summarized in Supplementary Table S3.

Sham-operated animals were subjected only to the surgical procedure to expose spinal cord without inducing injury. They were tested for BMS, TF reflex and immunohistochemical analysis concerning neuroglia and caspase (Supplementary Fig. S2).

### Micro computed tomography

Computed tomography datasets were acquired by a high-resolution 3D micro-CT imaging system (Skyscan 1172 G Bruker, Kontich – Belgium), with a camera pixel/size of 9 µm, 0.5 mm Al filter, tube voltage peak of 49 kV, tube current of 200 µA, exposure time of 250 ms and a scan time of 75 min. The 3D images were visualized using 3D Visualization Software CTvox v. 2.5 (Bruker). Details in^56^.

### Drugs

Saline or Docosahexaenoic acid (DHA – Sigma Aldrich) (0.082 mg/kg) were intraperitoneally (ip) administered within 1 hour from the surgery and repeated once/daily for 5 days. DHA dose was chosen on the basis of a previous study^13^.

### Behavioural tests

*BMS*. For injured animals the hindlimb locomotor function was assessed in an open field. Two raters blinded to the injury groups scored the functional performances of mice. The BMS score^23^ ranges from 0 to 9, where 0 indicates complete paralysis and 9 normal movement of the hindlimbs. Performances of the left and right hindlimbs were averaged in order to obtain the BMS score. Mice were tested for hindlimb functional deficits at 1, 2, 3, 4, 7, 10, 14, 17, 20, 30 days after SCI. Only mice completely paralysed (BMS scores = 0) after surgery were assigned to the experimental group classified as “severe” and used for the following analyses (n = 8/11 per group). The severity of spinal cord injury drastically influenced the survival of mice after surgery (Supplementary Table S3).

*TF*. As an additional way to check the hindlimb paralysis, we tested the withdrawal reflex to a thermal nociceptive stimulus by means of the tail-flick test (Ugo Basile), as previously described^57^.

### Immunohistochemistry

Seven, 30 or 60 days post-SCI, three or four mice from each experimental group were sacrificed for immunohistochemistry and perfused with saline followed by 4% paraformaldehyde in phosphate buffer saline (pH 7.4). Sections (40μ) of spinal cord tissue were prepared as previously described^54^. For double immunofluorescence staining, we used primary and secondary antibodies as in Supplementary Table S4. Nuclei were stained with DAPI (1:1000, Jackson ImmunoResearch).

### Confocal microscopy and quantification of immunoresponsivity

Images of immunostained sections were obtained by laser scanning confocal microscopy using a TCS SP5 microscope (Leica Microsystem). All analyses were performed in sequential scanning mode to rule out cross-bleeding between channels. Low (5X; 10X; 20X objective) and high magnification (63X) images of spinal cords sections were operated by I.A.S. software (Delta Systems, Italy). Quantification was performed using the ImageJ software (version 1.41, National Institutes of Health, USA).

Fluorescence of proteins was quantified by converting pixels in brightness values using the RGB method of the selected area. Positive cells (nuclei) were automatically counted with mark and count tool (n = 3 per group; 2/4 sections per animal, randomly selected and blinded analysed, except for the evaluation of gliosis in which n = 4/group were considered).

We examined the colocalization between NeuN or OLIG-1 and Caspase-3 by means of Manders’ coefficient^58^. This analysis uses an iterative procedure to determine what pair of thresholds for the 2 channels of the scatterplot gives a Manders’ correlation coefficient (r) of zero for the pixels below the thresholds (Evans, 34: 0–0.19 ‘very weak’, 0.20–0.39 ‘weak’, 0.40–0.59 ‘moderate’, 0.60–0.79 ‘strong’, 0.80–1.0 ‘very strong’). A double fluorescence image (red and green dyes) was splitted in RedGreenBlu channels and analysed. For each colocalization analysis, scatterplots were generated by the Image J program: the intensity of channel 1 pixels is used as the x-coordinate whereas the intensity of the channel 2 pixels as the y-coordinate.

For all groups, sections of ventral horns (VH), distal from the impact zone (3–5 mm rostro-caudal to lesion epicenter), in the peri-lesioned and in the injured area (epicenter T9–T11), were separately analysed. The lesion area and the formation of microcysts and cavity cavitation were evaluated by staining for glial fibrillary acidic protein (GFAP) 7, 30 and 60 days after SCI.

### Regenerated area and neuronal survival

The regenerated area was evaluated in longitudinal section of spinal cord (magnification 5X). Values have been obtained on the basis of the following formula: UA/RA*100, where UA is width of uninjured and RA the width of regenerated area. Neuronal survival was assessed by counting the NeuN-positive cells in the VH caudal to the lesion (L2).

### Gliosis evaluation

ImageJ quantification of GFAP expression was performed through Green pixels measurement converted in brightness values on longitudinal section of spinal cord (magnification 5X). Frame of rectangular areas of 100 μm for a total length of 800 μm (from epicenter vs caudal or cranial direction) was singularly examined. Damaged area (epicenter −100 μm to +100 μm) was not evaluated for absence of tissue.

### Morphometric analysis

Morphometric analysis was performed on high-resolution image acquisition criteria: 63X objective, zoom 3X, 1024 × 1024 frame, 10 Hz.

Each image was transformed in binary modality in order to obtain a digital image where outline of cell silhouettes were identified and automatically measured for astrocytes, while microglia were singularly counted and divided about the different morphology (R: Resting (ramified), H/B: Hyperactive/Bushy, U/A: Un-ramified/Ameboid).

### LC-MS experiments and bioinformatics analysis

Proteins extracted from SC tissue collected from saline- and DHA-treated groups (n = 3 mice/group) at day 7 were analyzed by shotgun proteomics experiments performed in LC-MS. Briefly, frozen spinal cord samples homogenized as described later for WB, were precipitated in a solution of 80% Acetone at −20 °C O.N. Precipitated proteins were resuspended in a dilution buffer compatible with trypsin digestion (6 M Urea in 100mMTris-Cl at pH7.8). Protein concentration was determined by Bradford Assay (BioRad) and 50ug of each sample digested with Trypsin, as previously described^59^, MClass nanoAcquity UPLC (Waters Co.) coupled with the Q TOF Synapt G2-Si (Waters Co.) were used for chromatographic separation of 0.25 ug of digested peptides and Data Independent MS analysis (MSe) as previously described^60^. Data from three replicate experiments for each pool were processed for qualitative and quantitative analysis using the software ProteinLynx Global Server v. 3.0.3 (PLGS, Waters)^59^, searching in a mouse database (UniProt KB/Swiss-Prot Protein Knowledgebase release 2016_09, released on October 15, 2016, restricted to Mus Musculus taxonomy) to which data from Saccharomyces cerevisiae Enolase (accession number: P00924) were appended.

Protein datasets were analysed through use of QIAGEN’s Ingenuity® Pathway Analysis (IPA®, QIAGEN Inc., https://www.qiagenbioinformatics.com/products/ingenuitypathway-analysis)^24^, Build version: 430520 M Content version: 31813283 (Release Date: 2016-12-05, www.ingenuity.com). The datasets, including the gene identifier and relative expression values (fold change DHA/SAL) of each protein, were uploaded for Core Analysis application that delivers a rapid assessment of the signaling and metabolic pathways, molecular networks, and biological processes that are most significantly perturbed.

### Western blot

For the myelin basic protein (MBP) analysis a total 5 µg of spinal cord samples (T9-T11), from 3 experimental groups (naïve, saline and DHA), at four time points (4 h-24 h-7 days-30 days) were used (n = 3 mice/group/time point). Membranes were incubated overnight at 4 °C with the following primary antibody: Myelin Basic Protein (MBP101, Abcam Cat# ab62631, RRID:AB_956157) and β-actin (AM1829b, Abgent Cat# AM1829b, RRID: AB_10664137). Antibody binding was revealed by using enhanced chemiluminescence (ECL) (Euroclone, Pero, Mi, Italy). Luminescent bands were imaged with autoradiography (X-ray) films (UltraCruz; Santa Cruz Biotechnolgy) and then scanning into a digital format. The βactin bands intensity was used as a control for equal protein loading and measured for densitometric analysis using ImageJ 1.49r software (Wayne Rasband, National Institutes of Health).

### Antibody array

The levels of various growth factors in spinal cord tissue lysates at 24 h and seven days (D7) post SAL and DHA treatment (n = 3 mice/group/time point) were analysed using a mouse antibody array (Mouse Cytokine Antibody Array G series; RayBiotech). Lysis buffer (Raybiotech) containing proteinase inhibitor cocktail (Sigma Aldrich) was added to homogenate impact zone fragments of spinal cords, and 30 µg of each sample was incubated with the array. Incubation and washes were performed according to the manufacturer’s instructions. The fluorescence intensity for each spot was detected using an Agilent G2564B microarray scanner (Agilent Technologies), quantified by the array testing services from RayBiotech and normalized to a positive internal controls on each array allowing the array comparison. The fold change (SAL/NAÏVE; DHA/NAÏVE) in each sample was then calculated and compared. According to the manufacturer’s instructions, any ≥1.5-fold increase or ≤0.65-fold decrease in signal intensity for a single analyte between samples may be considered a measurable and significant difference in expression.

### Statistics

Experimental data are expressed as mean ± SEM. Two-way ANOVAs for repeated measures were used to analyse BMS and TF results. Immunohistochemical data, Western blot, morphological analyses and antibody array were analysed by means of analysis of variance (ANOVA one-way), Kruskal-Wallis test or by Student’s T test depending on data dimension and number of variables. Post-hoc comparisons were carried using Tukey-Kramer test for ANOVA or Dunn’s Test (Bonferroni method) for Kruskal-Wallis. Data were considered statistically significant at p < 0.05. Statview SAS vers. 5.0, R and GraphPad Prism were used for data analysis.

## Supplementary information

Innovative mouse model mimicking human-like features of spinal cord injury: efficacy of Docosahexaenoic acid on acute and chronic phases.

Innovative mouse model mimicking human-like features of spinal cord injury: efficacy of Docosahexaenoic acid on acute and chronic phases

## Data Availability

The datasets generated during and/or analyzed during the current study are available from the corresponding author on reasonable request.
