# Publisher Correction: Innovative mouse model mimicking human-like features of spinal cord injury: efficacy of Docosahexaenoic acid on acute and chronic phases

**DOI:** 10.1038/s41598-019-53787-x

**Published:** 2019-11-14

**Authors:** Sara Marinelli, Valentina Vacca, Federica De Angelis, Luisa Pieroni, Tiziana Orsini, Chiara Parisi, Marzia Soligo, Virginia Protto, Luigi Manni, Roberto Guerrieri, Flaminia Pavone

**Affiliations:** 1 CNR - National Research Council, Institute of Cell Biology and Neurobiology, 00015 Monterotondo Scalo, Italy; 2 IRCCS - Santa Lucia Foundation, 00143 Roma, Italy; 3 CNR - National Research Council, Institute of Translational Pharmacology, 00133 Roma, Italy; 4 Department of Electrical, Electronic, and Information Engineering «Guglielmo Marconi», University of Bologna, 40136 Bologna, Italy

Correction to: *Scientific Reports* 10.1038/s41598-019-45037-x, published online 20 June 2019

In the original version of this Article, the author Federica De Angelis was incorrectly indexed. This error has now been corrected.

