# Supplementary material for: Innovative mouse model mimicking human-like features of spinal cord injury: efficacy of Docosahexaenoic acid on acute and chronic phases
Source: Sci Rep. 2019 Jun 20;9:8883. doi: 10.1038/s41598-019-45037-x (PMC6586623; doi:10.1038/s41598-019-45037-x)
Supplement: Supplementary file 1 — Innovative mouse model mimicking human-like features of spinal cord injury: efficacy of Docosahexaenoic acid on acute and chronic phases. [file 41598_2019_45037_MOESM1_ESM.pdf]

Innovative mouse model mimicking human-like features of spinal cord injury: efficacy of Docosahexaenoic acid on acute and chronic phases.

Sara Marinelli[1,2], Valentina Vacca[1,2], Federica De Angelis[2], Luisa Pieroni[2], Tiziana Orsini[1], Chiara Parisi[1], Marzia Soligo[3], Virginia Protto[3], Luigi Manni[3], Roberto Guerrieri[4], Flaminia Pavone[1,2*]

[1] CNR - National Research Council, Institute of Cell Biology and Neurobiology, 00015 Monterotondo Scalo ~~00143 Roma~~, Italy.
[2] IRCCS - Santa Lucia Foundation, 00143 Roma, Italy
[3] CNR - National Research Council, Institute of Translational Pharmacology, 00133 Roma, Italy.
[4] Department of Electrical, Electronic, and Information Engineering «Guglielmo Marconi», University of Bologna, 40136 Bologna, Italy

* To whom correspondence should be addressed: CNR – National Research Council, Institute of Cell Biology and Neurobiology, Via Ramarini 32, 00015 Monterotondo Scalo ~~Fosso di Fiorano 64, Roma 00143~~, Italy. Tel.: +39 06 50170 3189

Supplementary Information:

Supplementary Table S1: Cortical PinPoint Impactor parameters.
Supplementary Fig. S1: BMS scores and thermal threshold in female and male SCI mice
Supplementary Fig. S2: Behavioural and immunohistochemical analyses in sham female animals treated with saline or DHA.
Supplementary Table S2: Shotgun Proteomics
Supplementary Fig. S3: Bioinformatic analysis
Supplementary Fig. S4: WB analyses of p75, pJNK and JNK
Supplementary Table S3: Number of mice used in the SCI model
Supplementary Table S4: List of antibodies

Supplementary Table S1: Cortical PinPoint Impactor parameters corresponding to different degrees of SCI.

	TIPS	DWELL TIME	VELOCITY	DEPTH
MILD	Round 1-2	300/500 ms	3 m/sec	2,5-3 mm
MODERATE	Round 3	500/600 ms	3 m/sec	3,5-5 mm
SEVERE	Round 4	600/1000 ms	3 m/sec	5 mm

Supplementary Figure S1: (**A**) BMS scores and (**B**) thermal threshold measured in Tail-Flick test in female and male SCI mice (F and M respectively, n=8/each group) before (BL) and after a severe spinal trauma. Differently from females male mice gradually recovered after SCI.

A **B**

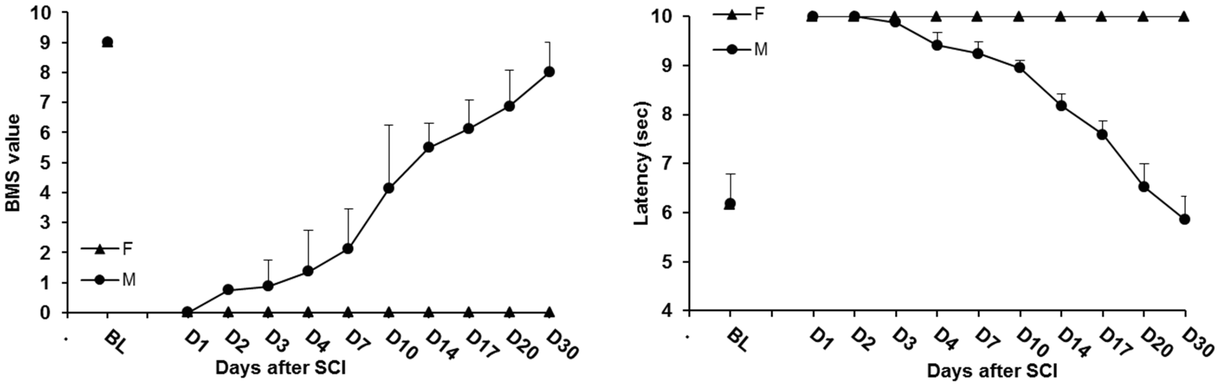

Supplementary Figure S2: Behavioural and immunohistochemical analyses in sham female animals treated with saline- (SAL) or DHA and compared with naïve mice. (**A)** BMS scores and (**B**) thermal threshold measured in Tail-Flick test in naïve (BL), SAL- and DHA-treated sham mice (**C**) Representative examples of high magnification (63x, zoom 2x) confocal images of astrocytes (GFAP, green) and microglia (IBA1, red). In graphs the dimension of astrocytes and the distribution of the different phenotypes of microglia are represented. In both saline- and DHA-treated sham mice no differences are observed in comparison with naïve animals. (**D**) Representative examples of high magnification (63x, zoom 2x) confocal images of oligodendrocytes (OLIG1, green) and caspase-3 (red). In graphs are reported brightness values of OLIG1 and Caspase-3 expression in ventral horns of spinal cord from naïve, SAL- and DHA-treated mice. In both saline- and DHA-treated sham mice no differences are observed in comparison with naïve animals. DAPI was used to stain nuclei.

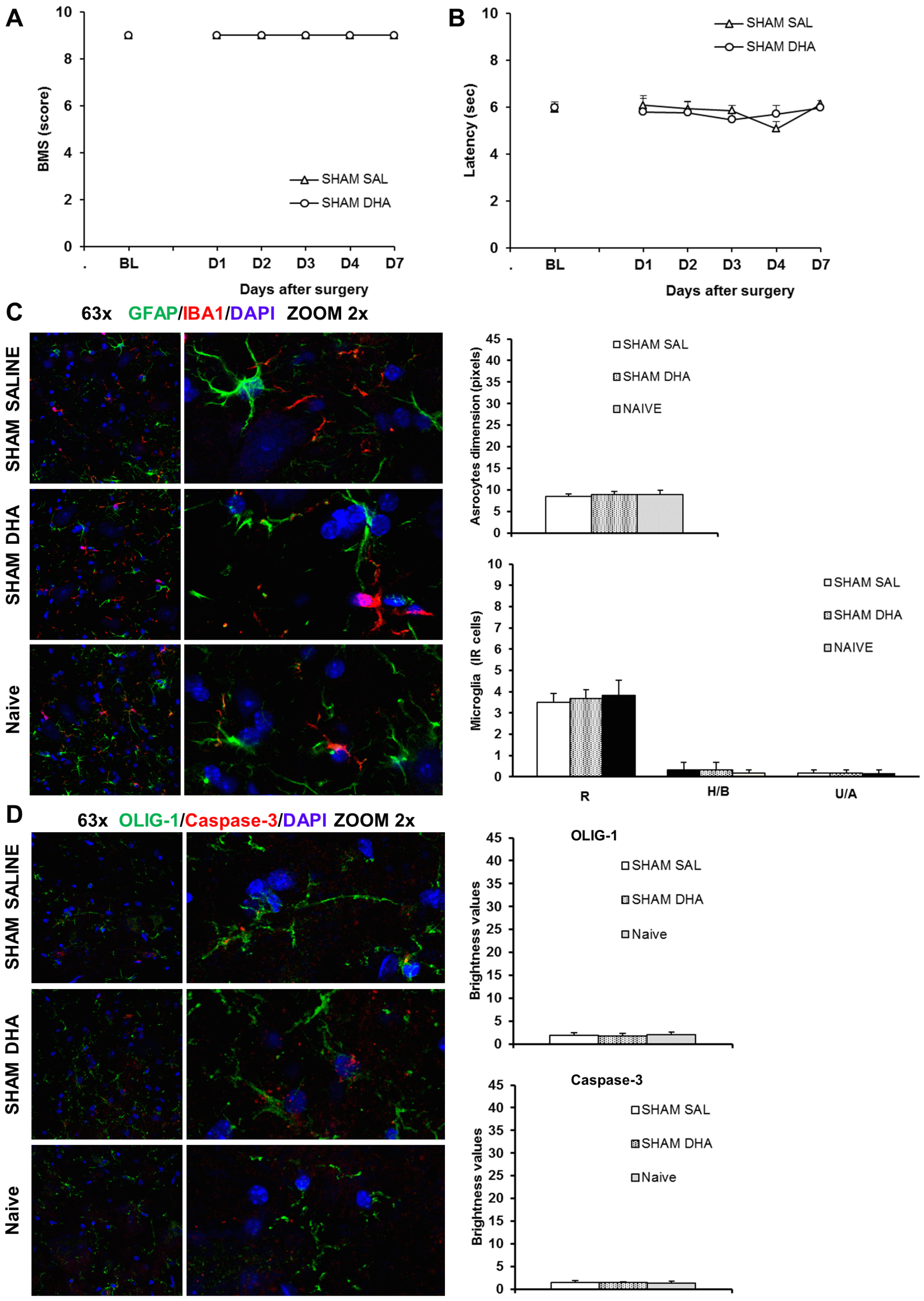

Supplementary Table S2. SHOTGUN PROTEOMICS: Differential Expression Analysis - DHA vs SALINE

(First part)

Accession*	Description	Gene Name	Score†	Unique‡	DHA: Saline	DHA:Saline _Log(e)Ratio
Accession*	Description	Gene Name	Score	Unique	DHA	DHA2
Q60932	Voltage-dependent anion-selective channel protein 1	Vdac1	949,08	DHA	DHA	DHA
P26645	Myristoylated alanine-rich C-kinase substrate	Marcks	567,62	DHA	DHA	DHA
P50518	V-type proton ATPase subunit E 1	Atp6v1e1	387,9	DHA	DHA	DHA
Q9WV92	Band 4.1-like protein 3	Epb41l3	375,98	DHA	DHA	DHA
Q6P8J7	Creatine kinase S-type_ mitochondrial	Ckmt2	987,49		2,18	0,78
Q91ZZ3	Beta-synuclein	Sncb	6237,11		2,08	0,73
P09041	Phosphoglycerate kinase 2	Pgk2	1446,45		1,93	0,66
Q9CRB6	Tubulin polymerization-promoting protein family member 3	Tppp3	2254,76		1,80	0,59
Q91V12	Cytosolic acyl coenzyme A thioester hydrolase	Acot7	809,99		1,80	0,59
O88935	Synapsin-1	Syn1	1864,8		1,77	0,57
O08553	Dihydropyrimidinase-related protein 2	Dpysl2	23417,02		1,72	0,54
P09411	Phosphoglycerate kinase 1	Pgk1	4803,99		1,68	0,52
Q9Z0F7	Gamma-synuclein	Sncg	474,84		1,67	0,51
P08249	Malate dehydrogenase_ mitochondrial	Mdh2	18052,8		1,65	0,50
Q7TQD2	Tubulin polymerization-promoting protein	Tppp	1554,69		1,63	0,49
Q8QZT1	Acetyl-CoA acetyltransferase_ mitochondrial	Acat1	874,11		1,63	0,49
Q62188	Dihydropyrimidinase-related protein 3	Dpysl3	6804		1,62	0,48
P17183	Gamma-enolase	Eno2	10918,73		1,62	0,48
Q9D3D9	ATP synthase subunit delta_ mitochondrial	Atp5d	3675,04		1,62	0,48
P09671	Superoxide dismutase [Mn]_ mitochondrial	Sod2	4153,61		1,58	0,46
Q01853	Transitional endoplasmic reticulum ATPase	Vcp	1012		1,58	0,46
P50114	Protein S100-B	S100b	2457,25		1,57	0,45
O55042	Alpha-synuclein	Snca	2544,23		1,55	0,44
P17742	Peptidyl-prolyl cis-trans isomerase A	Ppia	4620,59		1,54	0,43
Q9DCX2	ATP synthase subunit d_ mitochondrial	Atp5h	2877,1		1,52	0,42
Q99PT1	Rho GDP-dissociation inhibitor 1	Arhgdia	2683,34		1,52	0,42
P20108	Thioredoxin-dependent peroxide reductase_ mitochondrial	Prdx3	1010,04		1,52	0,42
P04370	Myelin basic protein	Mbp	22546,15		1,51	0,41
P30275	Creatine kinase U-type_ mitochondrial	Ckmt1	1436,43		1,49	0,40
Q8K183	Pyridoxal kinase	Pdxk	709,07		1,49	0,40
Q9ERD7	Tubulin beta-3 chain	Tubb3	11546,31		1,49	0,40
P56480	ATP synthase subunit beta_ mitochondrial	Atp5b	5201,2		1,49	0,40
P63038	60 kDa heat shock protein_ mitochondrial	Hspd1	2930,97		1,48	0,39
A2AQ07	Tubulin beta-1 chain	Tubb1	895,9		1,48	0,39
P21550	Beta-enolase	Eno3	5534,3		1,48	0,39
P52760	Ribonuclease UK114	Hrsp12	2664,1		1,46	0,38
P05202	Aspartate aminotransferase_ mitochondrial	Got2	3902,15		1,46	0,38
P05064	Fructose-bisphosphate aldolase A	Aldoa	12259,89		1,46	0,38
P99029	Peroxiredoxin-5_ mitochondrial	Prdx5	3426,36		1,46	0,38
Q8BWF0	Succinate-semialdehyde dehydrogenase_ mitochondrial	Aldh5a1	785,05		1,45	0,37
Q61171	Peroxiredoxin-2	Prdx2	3029,51		1,43	0,36
P17751	Triosephosphate isomerase	Tpi1	7191,61		1,43	0,36
P15105	Glutamine synthetase	Glul	1595,69		1,43	0,36
P02104	Hemoglobin subunit epsilon-Y2	Hbb-y	507,87		1,42	0,35
Q04447	Creatine kinase B-type	Ckb	7814,9		1,42	0,35
P97427	Dihydropyrimidinase-related protein 1	Crmp1	6625,2		1,39	0,33
P05063	Fructose-bisphosphate aldolase C	Aldoc	14216,55		1,39	0,33
Q9R0Y5	Adenylate kinase isoenzyme 1	Ak1	1038,8		1,38	0,32
Q9D051	Pyruvate dehydrogenase E1 component subunit beta_ mitochon	Pdhb	1095,84		1,38	0,32
P62204	Calmodulin	Calm1	12784,71		1,38	0,32
Q9DB20	ATP synthase subunit O_ mitochondrial	Atp5o	2931,77		1,38	0,32
Q9DBJ1	Phosphoglycerate mutase 1	Pgam1	5153,55		1,38	0,32
P10637	Microtubule-associated protein tau	Mapt	502,66		1,36	0,31
P70296	Phosphatidylethanolamine-binding protein 1	Pebp1	1977,02		1,36	0,31
Q8BH95	Enoyl-CoA hydratase_ mitochondrial	Echs1	879,08		1,36	0,31
P16858	Glyceraldehyde-3-phosphate dehydrogenase	Gapdh	30204,52		1,35	0,30

(Table S2 second part)

Accession*	Description	Gene Name	Score†	Unique‡	DHA: Saline	DHA:Saline Log(e)Ratio
Accession*	Description	Gene Name	Score	Unique	DHA	DHA2
O70456	14-3-3 protein sigma	Sfn	398,91		0,74	-0,30
P07901	Heat shock protein HSP 90-alpha	Hsp90aa1	777,96		0,70	-0,35
P50396	Rab GDP dissociation inhibitor alpha	Gdi1	1055,29		0,70	-0,36
Q61598	Rab GDP dissociation inhibitor beta	Gdi2	515,58		0,67	-0,40
P62259	14-3-3 protein epsilon	Ywhae	1722,86		0,67	-0,40
P16330	2'_3'-cyclic-nucleotide 3'-phosphodiesterase	Cnp	15261,35		0,64	-0,45
Q9Z1W8	Potassium-transporting ATPase alpha chain 2	Atp12a	484,54		0,64	-0,45
Q9CZU6	Citrate synthase_ mitochondrial	Cs	859,63		0,59	-0,52
P11499	Heat shock protein HSP 90-beta	Hsp90ab1	841,15		0,58	-0,54
Q8VDN2	Sodium/potassium-transporting ATPase subunit alpha-1	Atp1a1	905,94		0,53	-0,63
Q6PIC6	Sodium/potassium-transporting ATPase subunit alpha-3	Atp1a3	1404,93		0,52	-0,65
Q6PIE5	Sodium/potassium-transporting ATPase subunit alpha-2	Atp1a2	1215,46		0,51	-0,67
Q61885	Myelin-oligodendrocyte glycoprotein	Mog	1645,66		0,50	-0,70
Q8VDQ8	NAD-dependent protein deacetylase sirtuin-2	Sirt2	410,84		0,47	-0,75
P48962	ADP/ATP translocase 1	Slc25a4	565,74		0,47	-0,76
P0CG50	Polyubiquitin-C	Ubc	3502,35		0,44	-0,82
P14094	Sodium/potassium-transporting ATPase subunit beta-1	Atp1b1	893,95		0,44	-0,82
P0CG49	Polyubiquitin-B	Ubb	3502,35		0,44	-0,83
P62984	Ubiquitin-60S ribosomal protein L40	Uba52	3502,35		0,44	-0,83
P62983	Ubiquitin-40S ribosomal protein S27a	Rps27a	3502,35		0,44	-0,83
P07724	Serum albumin	Alb	5304,23		0,39	-0,95
P60202	Myelin proteolipid protein	Plp1	9752,25		0,21	-1,56
P18872	Guanine nucleotide-binding protein G(o) subunit alpha	Gnao1	564,5	Saline	Saline	
P14231	Sodium/potassium-transporting ATPase subunit beta-2	Atp1b2	888,79	Saline	Saline	
Q68FL4	Putative adenosylhomocysteinase 3	Ahcyl2	518,95	Saline	Saline	
P14211	Calreticulin	Calr	826,25	Saline	Saline	
Q60930	Voltage-dependent anion-selective channel protein 2	Vdac2	416,78	Saline	Saline	
P63001	Ras-related C3 botulinum toxin substrate 1	Rac1	1923,89	Saline	Saline	
Q9CZT8	Ras-related protein Rab-3B	Rab3b	726,65	Saline	Saline	
P61979	Heterogeneous nuclear ribonucleoprotein K	Hnrnpk	484,44	Saline	Saline	
Q9QYG0	Protein NDRG2	Nrdg2	532,43	Saline	Saline	
P43006	Excitatory amino acid transporter 2	Slc1a2	851,79	Saline	Saline	
P17710	Hexokinase-1	Hk1	503,87	Saline	Saline	
Q9D1G1	Ras-related protein Rab-1B	Rab1b	764,68	Saline	Saline	
Q05144	Ras-related C3 botulinum toxin substrate 2	Rac2	1736,33	Saline	Saline	
Q6PHN9	Ras-related protein Rab-35	Rab35	584,72	Saline	Saline	
Q921I1	Serotransferrin	Tf	1177,77	Saline	Saline	
Q80W21	Glutathione S-transferase Mu 7	Gstm7	562,85	Saline	Saline	
Q8R527	Rho-related GTP-binding protein RhoQ	Rhoq	705,04	Saline	Saline	
Q3UX10	Tubulin alpha chain-like 3	Tubal3	570,79	Saline	Saline	
P60766	Cell division control protein 42 homolog	Cdc42	732,17	Saline	Saline	
P60764	Ras-related C3 botulinum toxin substrate 3	Rac3	1580,35	Saline	Saline	
P13707	Glycerol-3-phosphate dehydrogenase [NAD(+)]_ cytoplasn	Gpd1	444,28	Saline	Saline	
P61028	Ras-related protein Rab-8B	Rab8b	510,17	Saline	Saline	
P61027	Ras-related protein Rab-10	Rab10	1171,43	Saline	Saline	
Q9ER71	Rho-related GTP-binding protein RhoJ	Rhoj	705,04	Saline	Saline	
P84096	Rho-related GTP-binding protein RhoG	Rhog	705,04	Saline	Saline	
P55258	Ras-related protein Rab-8A	Rab8a	510,17	Saline	Saline	
P62823	Ras-related protein Rab-3C	Rab3c	726,65	Saline	Saline	
P62821	Ras-related protein Rab-1A	Rab1A	510,17	Saline	Saline	
P08553	Neurofilament medium polypeptide	Nefm	763,84	Saline	Saline	
P08551	Neurofilament light polypeptide	Nefl	582,68	Saline	Saline	
Q80SW1	Putative adenosylhomocysteinase 2	Ahcyl1	563,85	Saline	Saline	

1. * Unique protein sequence identifier according to UniProtKB/Swiss-Prot Protein Knowledgebase, release 2016_09, October-05,2016 restricted to Mouse Taxonomy
2. † ProteinLynx Global Server score.
3. ‡ Protein found highly represented in the indicated group.

4. § Ratio of expression between two experimental groups.

Supplementary Figure S3: Bioinformatic analysis. Graphical representation predicted by Ingenuity Pathways Analysis (IPA, QIAGEN Inc., https://www.qiagenbioinformatics.com/products/ingenuitypathway-analysis) of the in out dataset (DHA vs SALINE). The relationships between molecules revealed by proteomics (described in Table 2) and other proteins are generated by the information contained onto a global molecular network enclosed in the Ingenuity Knowledge Base database.

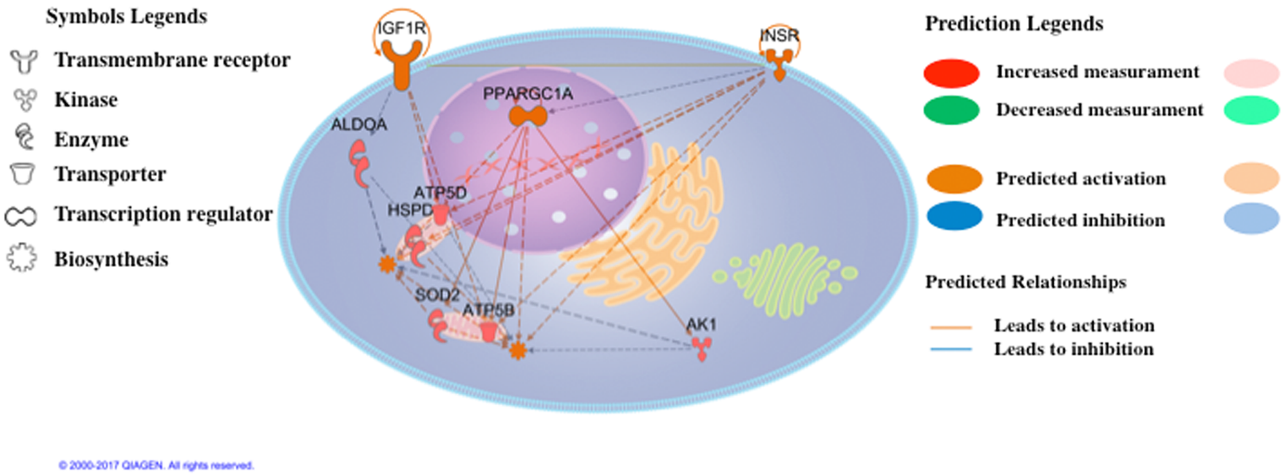

Supplementary Figure S4: WB analyses of **A**) p75 and **B**) pJNK and JNK, proteins involved in apoptotic pathway, in spinal tissues from naïve (N), saline- (S) and DHA-treated mice, analysed at the epicenter (E), peri-lesioned (P) and distal (D) areas. Both p75NTR and phosphorylated (p)-JNK were increased after SCI in the three spinal levels examined and were down-regulated by DHA °° p<0.001 °°°p<0.0001 vs naïve; ** p<0.001 vs SAL.

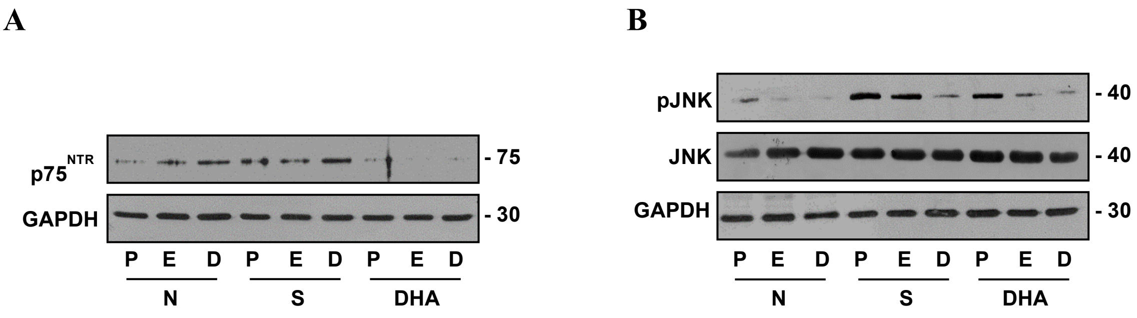

Methods. Western blot analysis was performed on tissue samples from different experimental groups. Tissue samples were homogenized in RIPA buffer, centrifuged at 4° C for 20 min at 13000 rpm, and then supernatants were stored at -20°C. 30µg of total protein spinal cord samples corresponding to T9-T11 (epicenter, E), T6-T8 (peri-lesioned, P) and L1-L3 (distal, D) were separated by 8% or 12% SDS-PAGE, and electrophoretically transferred to PVDF membrane overnight. Membranes were incubated overnight at 4°C with the following primary antibodies anti-p75NTR (Santa Cruz Biotechnology Cat# sc-53631, RRID:AB_784824); anti-GAPDH (Santa Cruz Biotechnology Cat# sc-25778, RRID:AB_10167668); anti-phospho-JNK (Cell Signaling Technology Cat# 9255, RRID:AB_2307321) and anti-JNK (Cell Signaling Technology Cat# 3708, RRID:AB_1904132). Horseradish peroxidase (HRP)-conjugated anti-rabbit or HRP-conjugated anti-mouse IgG was used as secondary antibody (Cell Signaling Technology). The blots were

developed with the enhanced chemiluminescence (ECL) detection system (WBKLS0500, Millipore). The GAPDH bands were used as a control for equal protein loading.

Supplementary Table S3: Number of mice used in the SCI model. In brackets is reported the number of dead mice. The severity of spinal cord injury drastically influenced the survival of mice after surgey: mild and moderate (Mod) contusion did not cause death, while severe (Sev) contusion induced death within 24h in about 44% of mice.

Treatment	Saline			DHA	
Contusion severity	Mild	Mod	Sev	Mild	Sev
EXPERIMENTS:					
BMS and TF	5 (0)	5 (0)	18 (7)	4 (0)	18 (10)
IF	-	-	5 (2)	-	5 (2)
WB	-	-	5 (2)	-	5 (2)
TOTAL	5 (0)	5 (0)	28 (11)	4 (0)	28 (14)

Supplementary Table S4: List of antibodies

	ANTIBODIES			
**PRIMARY**	**SPECIES**		**PRODUCT**	**MARKER**
NeuN	Mouse monoclonal	1:100	Millipore	Neurons
CD11b	Rat monoclonal	1:100	AbDSerotec	Microglia/macrophages
GFAP	Mouse monoclonal	1:100	Sigma-Aldrich	Astrocytes
OLIG1	Mouse monoclonal	1:100	Santa Cruz	Oligodendrocyte
Caspase-3	Rabbit polyclonal	1:100	Cell signaling	apoptosis
proNGF	Rabbit polyclonal	1:100	Chemicon	Pro-regeneration/apoptosis
**SECONDARY**	**SPECIES**		**PRODUCT**	**FLUORESCENCE**
Alexa Fluor 488	Donkey anti- mouse	1:100	Jackson ImmunoResearch	Green
Cy2	Donkey anti-rat	1:100	Jackson ImmunoResearch	Green
Rhodamine	Goat anti-rabbit	1:100	Jackson ImmunoResearch	Red
